# Comparative transcriptome combined with metabolome analyses revealed key factors involved in nitric oxide (NO)-regulated cadmium stress adaptation in tall fescue

**DOI:** 10.1186/s12864-020-07017-8

**Published:** 2020-08-31

**Authors:** Huihui Zhu, Honglian Ai, Zhengrong Hu, Dongyun Du, Jie Sun, Ke Chen, Liang Chen

**Affiliations:** 1 College of Resources and Environmental Science, South-Central University for Nationalities, Wuhan, P.R. China; 2 CAS Key Laboratory of Plant Germplasm Enhancement and Specialty Agriculture, Wuhan Botanical Garden, Chinese Academy of Sciences, Wuhan, P.R. China; 3 College of Pharmacy, South-Central University for Nationalities, Wuhan, P.R. China; 4 Center of Economic Botany, Core Botanical Gardens, Chinese Academy of Sciences, Wuhan, P.R. China

**Keywords:** Tall fescue, Cd stress, Nitric oxide, RNA-Seq, Metabolite profiling, Detoxification mechanism

## Abstract

**Background:**

It has been reported that nitric oxide (NO) could ameliorate cadmium (Cd) toxicity in tall fescue; however, the underlying mechanisms of NO mediated Cd detoxification are largely unknown. In this study, we investigated the possible molecular mechanisms of Cd detoxification process by comparative transcriptomic and metabolomic approaches.

**Results:**

The application of Sodium nitroprusside (SNP) as NO donor decreased the Cd content of tall fescue by 11% under Cd stress (T1 treatment), but the Cd content was increased by 24% when treated with Carboxy-PTIO (c-PTIO) together with Nitro-L-arginine methyl ester (L-NAME) (T2 treatment). RNA-seq analysis revealed that 904 (414 up- and 490 down-regulated) and 118 (74 up- and 44 down-regulated) DEGs were identified in the T1 vs Cd (only Cd treatment) and T2 vs Cd comparisons, respectively. Moreover, metabolite profile analysis showed that 99 (65 up- and 34-down- regulated) and 131 (45 up- and 86 down-regulated) metabolites were altered in the T1 vs Cd and T2 vs Cd comparisons, respectively. The integrated analyses of transcriptomic and metabolic data showed that 81 DEGs and 15 differentially expressed metabolites were involved in 20 NO-induced pathways. The dominant pathways were antioxidant activities such as glutathione metabolism, arginine and proline metabolism, secondary metabolites such as flavone and flavonol biosynthesis and phenylpropanoid biosynthesis, ABC transporters, and nitrogen metabolism.

**Conclusions:**

In general, the results revealed that there are three major mechanisms involved in NO-mediated Cd detoxification in tall fescue, including (a) antioxidant capacity enhancement; (b) accumulation of secondary metabolites related to cadmium chelation and sequestration; and (c) regulation of cadmium ion transportation, such as ABC transporter activation. In conclusion, this study provides new insights into the NO-mediated cadmium stress response.

## Background

Cadmium (Cd) is considered as one of the most toxic metals to all living organisms. Cd is derived from various sources, and the anthropogenic activities have accelerated the release of Cd into the surrounding environment [1–3]. More importantly, Cd is highly soluble and mobile which gives it easy access to the food chain, and subsequently causes serious harm to human health [4–7]. Thus, adequate understanding of the plants regulatory mechanism under Cd stress becomes an urgent issue. When plants are exposed to cadmium stress, the cellular redox homeostasis is disrupted, leading to the production and burst of reactive oxygen species (ROS) in plant cells [8, 9]. Accordingly, various genes and metabolites are regulated and processes including antioxidant system, pathogen defense, detoxification mechanism, programmed cell death, and stomatal behavior are evoked [10].

Nitric oxide (NO), a free radical reactive gas, is a multifunctional signaling molecule [11–13]. NO plays crucial roles in many plant physiological processes, such as, germination, lateral root development, photosynthesis, phytohormone modulation, and cell death and growth [14–16]. The role of NO and ROS crosstalk has been known to be in the regulation of new adventitious roots (NARs) formation and primary root biomass accumulation (PRBA) under arsenate stress [17]. Many studies have shown that NO plays critical roles in plant stress response through the modulation of protein kinase and antioxidant enzymes activities, the mobilization of flavonoids and other secondary metabolites, such as SA, ET, IAA, ABA and JA, and the activation of the expression of related genes encoding ABC transporter, GSTs and cytochromes P450 to amplify Cd uptake [2, 16, 18–20]. Our previous research revealed that NO alleviated Cd^2+^ toxicity on the PSII electron donor side [21]. Although the significance of NO in plant heavy metal stress response is established, the interaction between Cd stress and NO signaling as well as more comprehensive molecular and metabolic mechanisms of the NO-mediated alleviation of Cd stress are still unclear.

Tall fescue (*Festuca arundinacea Schreb*), is one of the most commonly used cool season forage and turf grass species worldwide. It has a well-developed root system with a broad adaptability to various environmental changes [21]. In recent studies, tall fescue shows high tolerance as well as enrichment ability to various heavy metals, including Cd, Cu, Pb and Zn [22–24]. Tall fescue can potentially be applied as an accumulator species in Cd-accumulated soil for phytoremediation. Our previous study revealed that the genes encoding glutathione S-transferase (GST), transporter proteins, pathogenesis/disease-related proteins and transcription factors were significantly induced to respond to Cd stress in roots, when tall fescue was exposed to Cd stress [25]. It has been reported that NO application can alleviate Cd^2+^ toxicity in tall fescue [21]; however, the role of endogenous NO against Cd stress in tall fescue is still not well understood [26]. Therefore, it is important to explore the comprehensive molecular and metabolic mechanisms of the NO-mediated alleviation of Cd stress in tall fescue. Here, comparative metabolomic and transcriptomic analyses were performed to elucidate the underlying mechanisms of the NO-regulated Cd detoxification process in tall fescue. Our data suggest NO play a significant role in the alleviations of Cd-induced stress. This study provides new insights into the NO-regulated Cd stress response in tall fescue.

## Results

### NO supplementation decreases cadmium accumulation

**Fig. 1:**
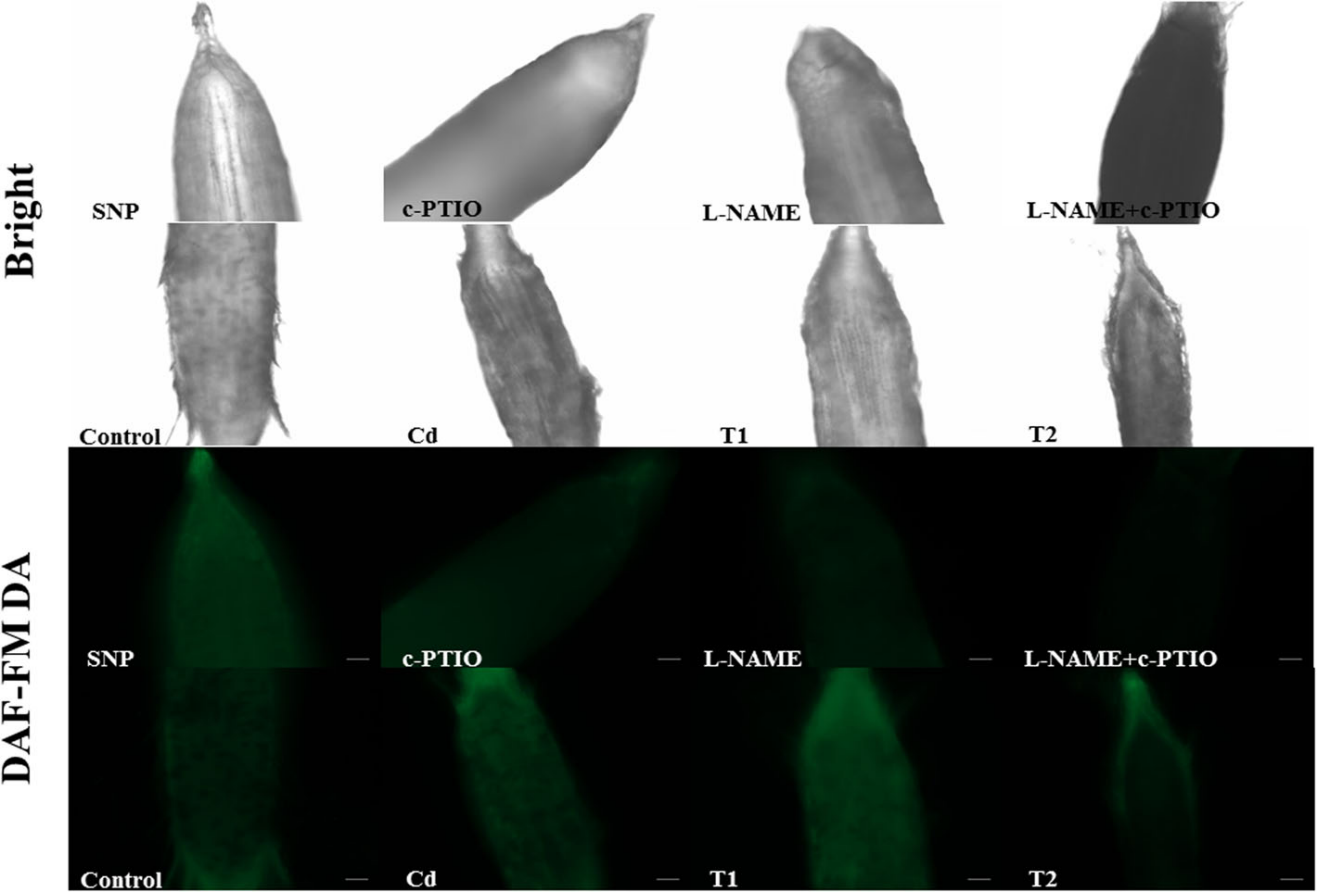
The NO content in tall fescue roots. Tall fescue roots were loaded with the sensitive fluorescent dye DAF-FM DA. **a** and **b** represent bright and fluorescent field, respectively. The lower corner of each photo has a scale bar in 50 μm length

**Fig. 2:**
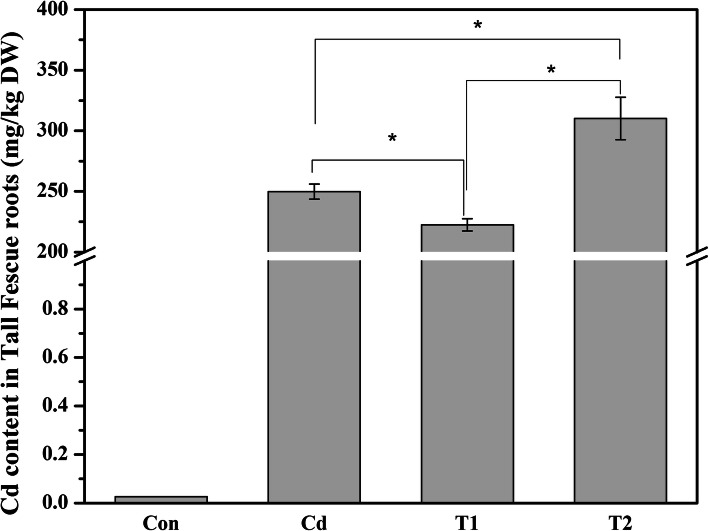
The Cd content in roots of tall fescue under four different treatments. There were four regimes, comprising Control (Con), Cd, T1 and T2 treatment. Values are given as mean ± SE (*n* = 3). Data about Cd, T1 and T2 treatment were analyzed using one-way Analysis of Variance, followed by LSD test. Asterisks (*) indicate the significant difference at *P* < 0.05

To investigate the efficacy of different treatments used in the study, NO contents were measured (Fig. 1, Table 1, Table S1, S2 and S3). There was a significant difference in NO contents between roots treated with or without Cd (Cd level) by a two-way analysis of variance and LSD test. A significant difference was also found at NO level among groups with addition of NO donor (SNP and T1 treatment), NO production inhibitor and NO scavenger (L-NAME+c-PTIO and T2 treatment) and without the addition of NO donor, inhibitor or scavenger (Control and Cd treatment). However, there was no significant difference at the Cd × NO level (Table 1). NO contents in the roots treated by L-NAME plus c-PTIO were significantly lower than those of Control (Table S1, Table S3). By contrast, SNP alone treatment significantly increased NO content in roots compared to Control. Moreover, NO contents in the roots were significantly increased in plants under Cd treatment compared to Control. NO accumulation was further increased when plants were treated with Cd plus NO donor SNP (T1), while it was obviously reduced in roots exposed to Cd, L-NAME plus c-PTIO (T2), compared to Cd treatment, respectively. These results suggested the above NO-related reagent treatment and Cd treatment could affect endogenous NO contents of tall fescue root effectively. To further investigate NO-regulated cadmium stress adaptation in tall fescue, we focused on the four treatment groups including Control, Cd, T1 and T2. The Cd content was reduced in tall fescue roots by 11% in T1 regime relative to Cd sole treatment (Fig. 2). On the contrary, the Cd content was markedly increased by 24.2% when plants were treated with c-PTIO and L-NAME. Besides, there was no significant difference in root dry weight and root length among Cd, T1 and T2 treatment group that may be due to short-time treatment (48 h) (data not shown). These results indicated that NO can effectively decrease the Cd accumulation in the tall fescue roots.

**Table 1:** The result of two-way Analysis of Variance about relative fluorescence intensity in tall fescue roots except L-NAME treatment and c-PTIO treatment

Source of variation	Sum of Squares	df	Mean Square	F	*P*-value	F_0.05_
NO level	10.5757	2	5.2879	72.2668	0.0000	3.5546
Cd level	1.5019	1	1.5019	20.5265	0.0003	4.4139
Cd × NO level	0.0704	2	0.0352	0.4811	0.6258	3.5546
Error	1.3171	18	0.0732			
Total	13.4652	23

### Transcriptome analysis

**Fig. 3:**
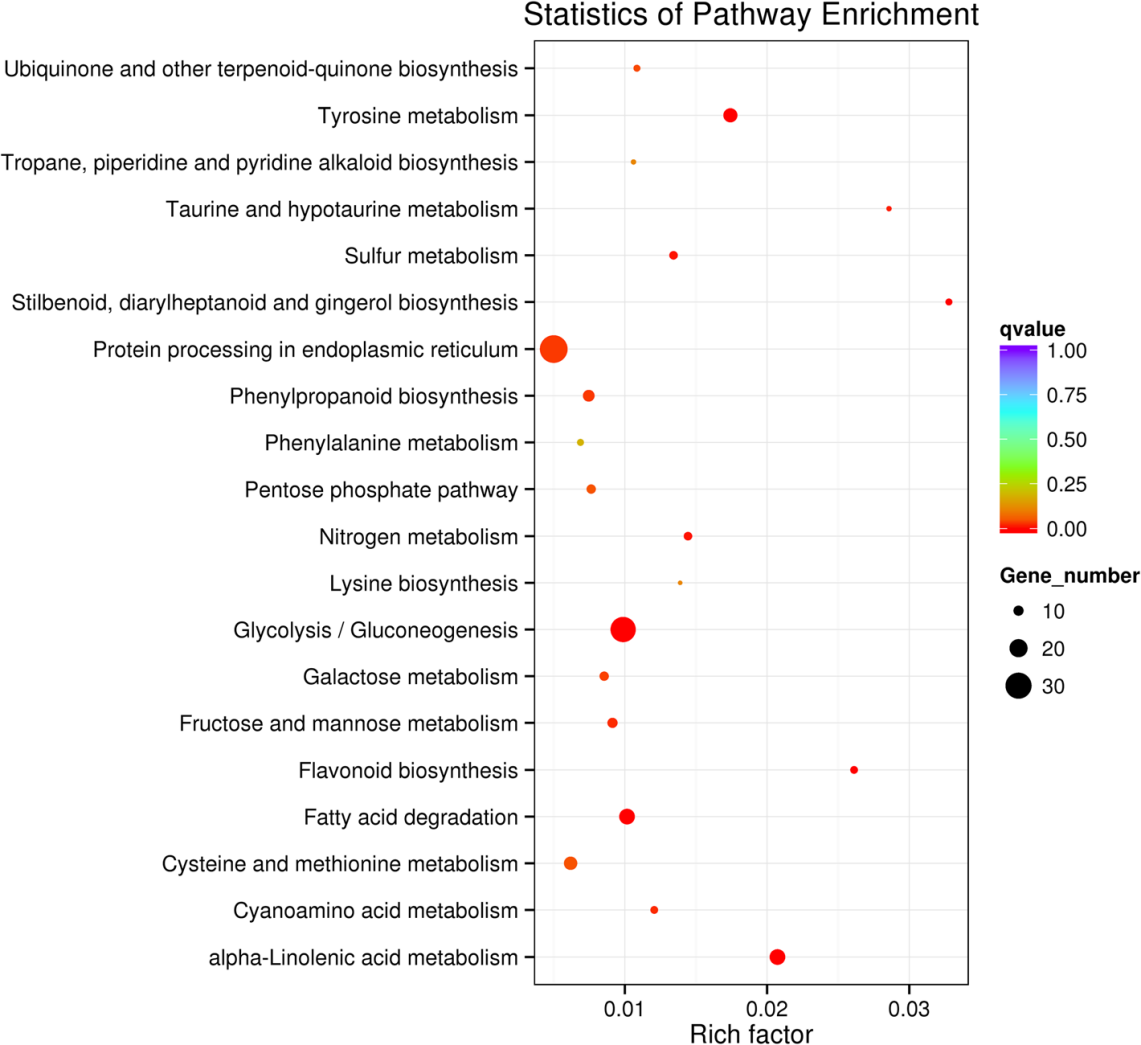
Scatter plot analysis of the DEGs in response to the T1 treatment in tall fescue roots. The tall fescue seedlings were cultivated in 1/2 Hoagland solution with 50 mg/L Cd^2+^ (CdCl_2_•2.5H_2_O) (Cd treatment) and 1/2 Hoagland solution with 50 mg/L Cd^2+^ and 200 μM SNP (T1 treatment), respectively. Each value is the mean of three replicates

To investigate the molecular mechanisms of Cd detoxification by NO in tall fescue, transcriptome analysis was performed. A total of 12 RNA-seq libraries were constructed and sequenced to identify the DEGs that were responsive to Cd stress in the tall fescue roots with or without NO treatment. Table S4 showed an overview of the RNA-Seq reads obtained from the 12 libraries. A total of 2,005,577 transcripts were retrieved from the clean reads. The length of the transcripts ranged from 201 bp to 16,784 bp, and the mean length was 680 bp. A total of 968,924 unigenes were obtained from these transcripts, and the mean length was475 bp with an N50 length of 560 bp (Table S5). Based on de novo assembly, a total of 904 DEGs (414 up-regulated and 490 down-regulated) were identified in the T1 vs Cd comparison, but only 118 DEGs (74 up- and 44 down-regulated) were identified in the T2 vs Cd comparison (Fig. S1). Additionally, we found that 1482 genes (591 up-regulated and 891 down-regulated genes) were differentially expressed in the Cd vs Control comparison. Meanwhile, to confirm the reliability of the Illumina RNA-Seq, some DEGs involved in different biological processes were selected and detected by quantitative reverse transcription-PCR (qRT-PCR) analysis. The strong correlation between the result from qRT-PCR and RNA-Seq (r = 0.8935) indicated that the RNA-Seq was accurate and effective as shown in Fig. S8. GO functional and enrichment analyses were performed to classify the functions of DEGs (Fig. S2). The results demonstrated that biological processes (BP) were the most enriched among the GO categories, and metabolic processes formed the dominant group in this category. The molecular function (MF) was the second most enriched, and the highly represented GO terms were oxidoreductase activity, metal ion binding and cation binding. The cellular components (CC) were the least enriched category in the GO classification. Furthermore, the number of DEGs was significantly enriched using the KEGG database compared to the background number (q < 0.05). The enriched KEGG pathways were presented using a scatterplot method (Fig. 3). According to their enrichment factor, the top-eight enriched pathways were related to “stilbenoid, diarylheptanoid and gingerol biosynthesis”, “taurine and hypotaurine metabolism”, “flavonoid biosynthesis”, “alpha-linolenic acid metabolism”, “tyrosine metabolism”, “nitrogen metabolism”, “lysine biosynthesis” and “sulfur metabolism” from largest to smallest.

### Metabolomics

**Fig. 4:**
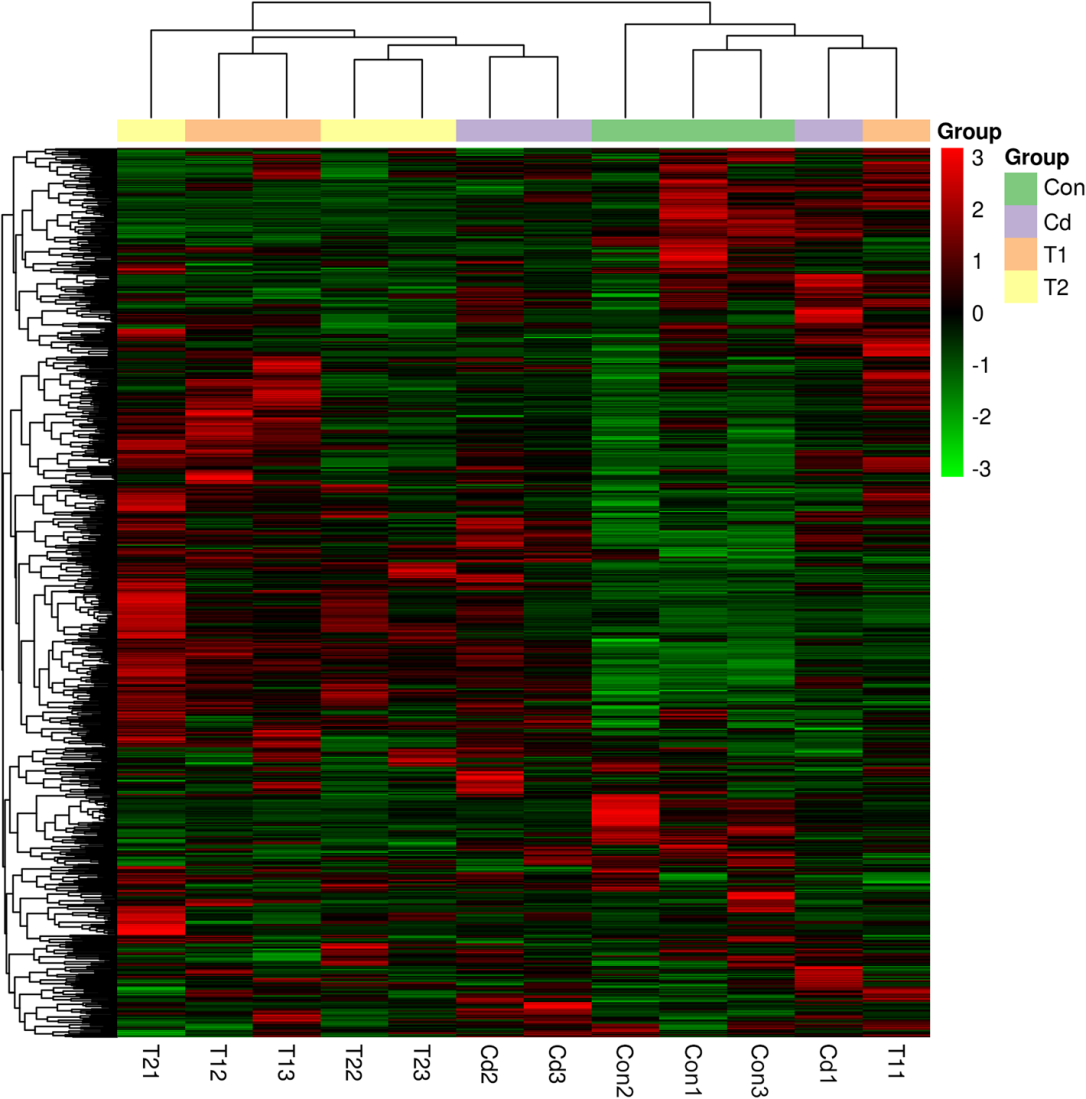
The hierarchical cluster analysis (HCA) of the metabolite profiles in tall fescue. There were four treatment regimes in this study, including the Con, Cd treatment, T1 treatment, and T2 treatment, and each regime had three replicates. They respectively presented the tall fescue seedlings were cultivated in 1/2 Hoagland solution (Con), 1/2 Hoagland solution with 50 mg/L Cd^2+^ (CdCl_2_•2.5H_2_O) (Cd treatment), 1/2 Hoagland solution with 50 mg/L Cd^2+^ and 200 μM SNP (T1 treatment), and 1/2 Hoagland solution with 50 mg/L Cd^2+^, 200 μM L-NAME and 100 μM c-PTIO (T2 treatment)

Gas chromatography/time-of-flight mass spectrometry (GC-TOF-MS) was performed to identify the differentially expressed metabolites modulated by exogenous NO in tall fescue under Cd stress. A total, 823 metabolites were detected in T1 vs Cd comparison. A total of 99 metabolites, mainly comprising amino acids and their derivatives (14), flavones (18), anthocyanins (11), phenol amides (7), organic acids and their derivatives (4), sugars (3) and others (42), showed a significant response to the T1 treatment compared with the Cd treatment (VIP ≥ 1, fold change≥2 or fold change≤0.5) (Table S6). For these differentially expressed metabolites, 65 metabolites were up-regulated and 34 metabolites were down-regulated. Besides, 131 differentially expressed metabolites (45 up- and 86 down-regulated) were identified in the T2 vs Cd group and 197 differentially expressed metabolites (161 up- and 36 down-regulated) were found in the Cd vs Control group. Moreover, the majority of the compounds detected were altered in response to the NO treatment (Fig. S3), and the most prominent group was the secondary metabolites, particularly anthocyanin and flavone. In the T1 vs Cd comparison, the metabolites peonidin (Fes0607) and rhoifolin (Fes1007) were up-regulated at levels 360.7- and 99.9-fold higher, respectively. However, the peonidin down-regulation was 2525-fold higher in the Cd vs Control group. On the other hand, the flavone metabolites acacetin (Fes1079) and sakuranetin (Fes1078) were largely down-regulated in the T1 vs Cd comparison (Table S7). Principal component analysis (PCA) revealed that the first and second principal components accounted for 27.63 and 14.27% of the total variance, respectively (Fig. S4). Furthermore, the first principal component showed a separation of the Control treatment and the treatments containing Cd (including Cd, T1 and T2). In addition, the second principal component implied that the sample was treated by exogenous NO or inhibited to produce NO. The analysis of the metabolite profiles by PCA revealed a clear separation of all treated samples. Simultaneously, the results of the hierarchical cluster analysis (HCA) suggested that all the Control samples clustered as a single distinct group (Fig. 4). In contrast, the other treatment lines did not form a single cluster. It is important that the two Cd lines and the two T2 lines clustered in the same manner, suggesting that NO level may be lower and closer to the Cd treatment in the large category.

### Overview of the correlations in metabolomics and transcriptomics

**Fig. 5:**
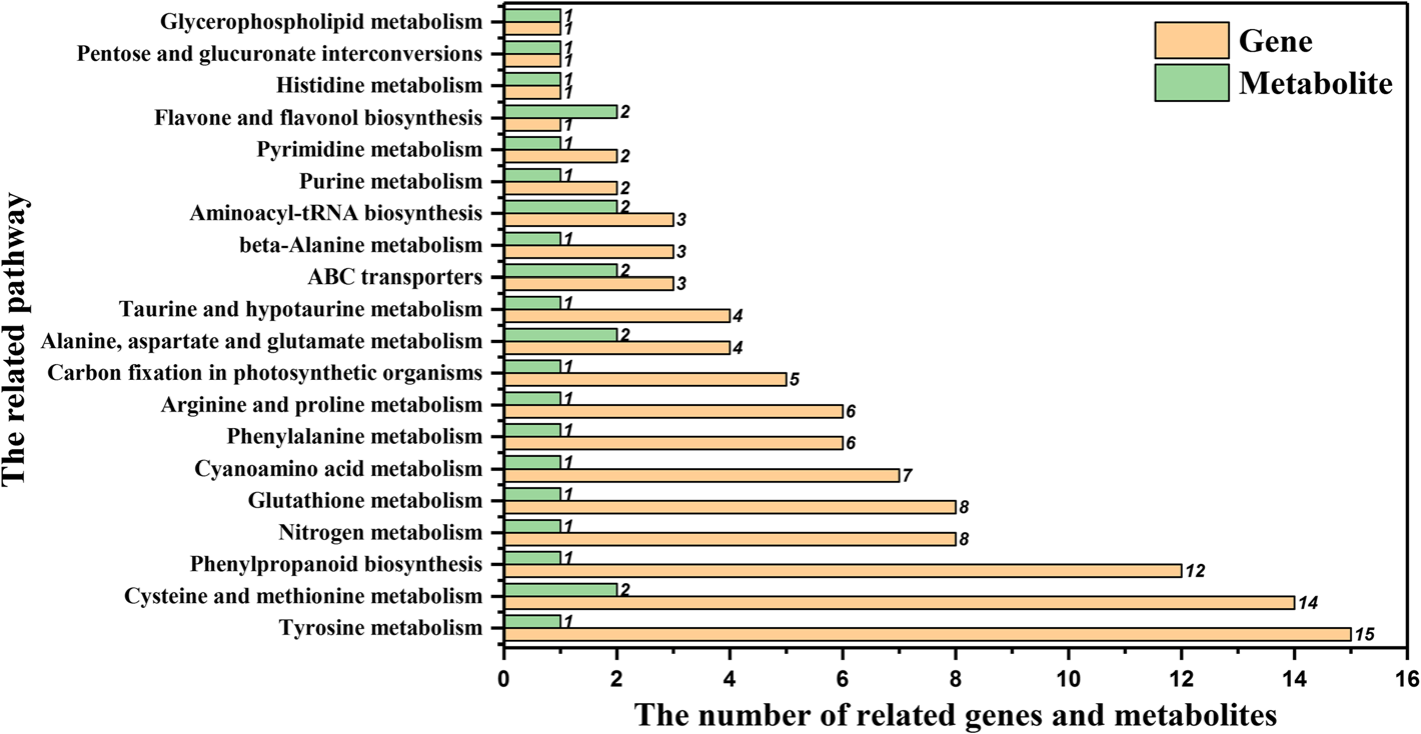
Histogram of the differentially expressed related genes and metabolites in response to the T1 treatment vs Cd treatment in tall fescue. The tall fescue seedlings were cultivated in 1/2 Hoagland solution with 50 mg/L Cd^2+^ (CdCl_2_•2.5H_2_O) (Cd treatment) and 1/2 Hoagland solution with 50 mg/L Cd^2+^ and 200 μM SNP (T1 treatment), respectively. Each value is the mean of three replicates

Correlation analysis was performed between metabolomics and transcriptomics data to further reveal the role of NO in plant Cd stress response. By comprehensive integrated analysis of all data with or without NO treatment, the emphasis was given to related genes and metabolites between T1 and Cd treatment. The integrated analysis of the transcriptome and metabolome between T1 and Cd treatment demonstrated that 81 out of 904 DEGs showed enriched correlations with metabolites and mainly included GSTs, nitrate reductase (NAD(P)H), trans-cinnamate 4-monooxygenase, and ABC transporters. In addition, 255 of the metabolites detected were enriched, but only 15 metabolites were differently expressed. The data for the annotated metabolites were presented as a heat-map (Fig. S5). Three clusters were informative concerning the differential NO response under Cd stress. The metabolites in the middle cluster were mainly involved in amino acids, especially L-pyroglutamic acid (Fes0791). They showed higher levels under the T1 treatment than the Cd treatment. The differently expressed genes and metabolites were enriched in the same KEGG pathways, including phenylpropanoid biosynthesis, nitrogen metabolism, flavone and flavonol biosynthesis, and ABC transporters (Fig. 5, Fig. S6 and Table S8). The DEGs were mainly involved in antioxidant systems, secondary metabolic pathways, nitrogen metabolism and metal ion transport mechanism, suggesting that NO alleviated Cd stress by a wide series of defense mechanisms.

A correlation between the transcripts and metabolites was analyzed for the transcripts related to the secondary metabolic pathways, such as those for phenylpropanoid biosynthesis (trans-cinnamic acid), flavone biosynthesis (isotrifoliin and acacetin) and organic acids (2, 5-dihydroxybenzoic acid). On the other hand, correlations were found for pathways related to nitrogen metabolism and some amino acid biosynthesis (L-citrulline, L-pyroglutamic acid and L-alanine).

**Fig. 6:**
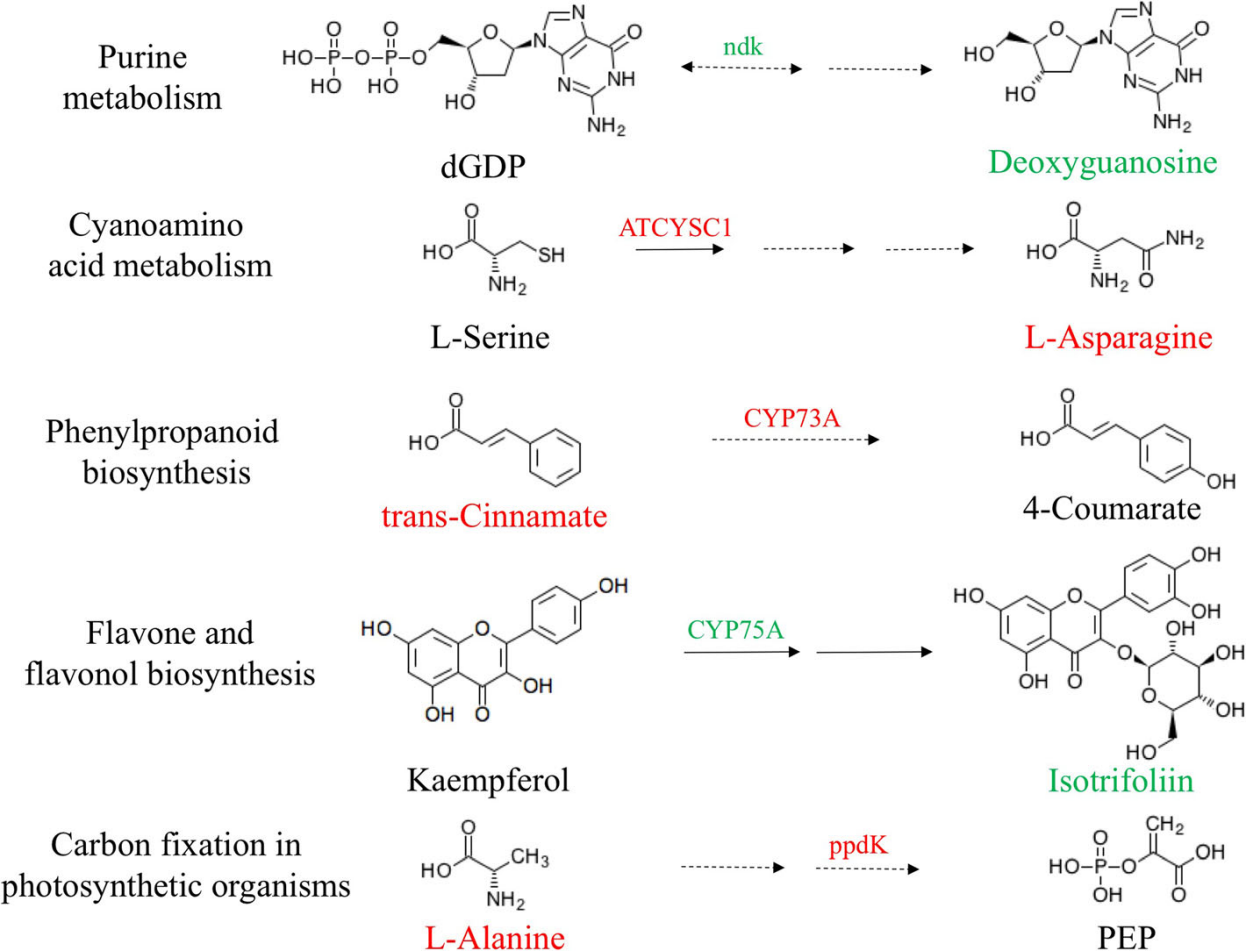
The simplified schematic of the related region of some pathways in the T1 treatment vs Cd treatment in tall fescue. The tall fescue seedlings were cultivated in 1/2 Hoagland solution with 50 mg/L Cd^2+^ (CdCl_2_•2.5H_2_O) (Cd treatment) and 1/2 Hoagland solution with 50 mg/L Cd^2+^ and 200 μM SNP (T1 treatment), respectively. The green labels show the down-regulated genes or metabolites, and the red labels show the up-regulated genes or metabolites. The solid arrows indicate that the biosynthesis of the metabolite indicated is a single approach. The dotted arrows indicate the biosynthesis of the metabolite indicated is a multipath

To understand the interaction between differentially expressed genes and metabolites more clearly, we selected some related DEGs mapped into the related metabolic pathways. In the T1 vs Cd comparison, correlations were observed for pathways, such as phenylpropanoid biosynthesis and flavone and flavonol biosynthesis (Fig. 6). The transcripts related to phenylpropanoid biosynthesis showed the highest negative correlations with the metabolite trans-cinnamic acid. In the region from trans-cinnamic acid to 4-coumarate, trans-cinnamic acid and the genes encoding CYP73A, located in the downstream of trans-cinnamic acid, were up-regulated. The up-regulated content of trans-cinnamic acid may contribute to the increased transcription of CYP73A. It has been reported that trans-cinnamic acid improved the growth of plants and showed an inverse effect compared to the plants treated with heavy metals (Hojati et al., 2016). Additionally, some interrelated DEGs (TRINITY_DN367754_c1_g1, TRINITY_DN359843_c1_g1, TRINITY_DN359843_c2_g1, TRINITY_DN380055_c1_g4 and TRINITY_DN380055_c1_g2) were up-regulated not only in the T1 vs Cd condition but also in the T2 vs Cd condition. Strikingly, the level of isotrifoliin increased more than 4-fold in the T2 vs Cd comparison, whereas the opposite trend was observed in the T1 vs Cd comparison. Meanwhile, the gene encoding CYP75A, located in the upstream of isotrifoliin, was down-regulated in the T1 vs Cd comparison. However, the gene encoding CYP75A was not changed in the T2 vs Cd comparison. Therefore, we speculated that the down-regulated expression of CYP75A gene caused the decline of isotrifoliin indirectly. Furthermore, the organic acid 2, 5-dihydroxybenzoic acid showed similar expression patterns like isotrifoliin. All the related genes regarded ADH1, an alcohol dehydrogenase, which catalyzes the conversion of pyruvate to ethanol. Additionally, all of the ADH1-related genes were up-regulated, but only the gene encoding the flavonoid 3′, 5′-hydroxylase (CYP75A) was down-regulated in the T1 treatment.

There is no doubt that the SNP application will be involved in nitrogen metabolism. Additionally, it has been reported that SNP application could protect the plants against abiotic stress through its impact on nitrogen metabolism. Therefore, it was obvious to find a correlation between metabolome and transcriptome for nitrogen metabolism in T1 treatment. In this case, it was obvious that nitrate reductase (TRINITY_DN380554_c0_g2, TRINITY_DN348032_c3_g1 and TRINITY_DN367739_c0_g2), as the first identified NO biosynthetic enzyme, were up-regulated in the T1 vs T2 condition, and the concentration of L-citrulline (the NO-accompanying product) was increased compared with that of the Cd treatment. Some genes involved in NO-generating mechanisms were also changed. The gene encoding arginase was down-regulated, and some nitrate or nitrite transporter (NRT) were apparently up-regulated. Similarly, two transporter members of the ABC chaperone family (ABCB1 and ABCC10) were significantly up-regulated.

## Discussion

In the present study, transcriptomics and metabolomics data were integrated to investigate the molecular mechanisms of NO-mediated Cd detoxification in tall fescue. A total of 81 DEGs and 15 differently related metabolites were screened out from the T1 vs Cd comparison. The results showed that specific genes and metabolites were induced to counteract the cadmium stress following NO treatment. Moreover, the metabolites were mainly involved in defense mechanism, such as increasing antioxidant capacity, excreting more secondary metabolites for Cd chelation and sequestration, and regulating some transporters to decrease the absorption of cadmium as shown in Fig. 2.

### The mechanisms of improving the antioxidant capacity

The mechanisms of the oxidant system are tightly connected to Cd toxicity [25, 27–29]. Plants subjected to heavy metal stresses generate ROS, which causes the oxidative damage to cellular structures and functions [28, 30, 31]. Nitric oxide is reported to play a role in regulating the antioxidant system [32]. In our study, a large number of ROS-related terms were identified and grouped into significantly and differentially expressed GO terms, including response to oxidative stress, oxidation-reduction processes and peroxidase activity. Additionally, the activities of oxidative enzymes, including SOD and APX, suggested that NO plays important roles in regulating the antioxidant system (Fig. S7). Furthermore, genes and metabolites, especially genes encoding enzymes such as peroxidase, glutamate decarboxylase, glucose-6-phosphate 1-dehydrogenase and glutathione S-transferase which are related to oxidative stress response showed significant changes after SNP treatment under Cd stress. Additionally, most of the flavone-related metabolites acting as non-enzymatic antioxidants were up-regulated in the T1 vs Cd comparison (Table S6). In fact, many previous studies showed a close connection between NO and genes related to antioxidant enzymes, such as GSTs [33, 34]. In addition, the expression patterns of glutathione transferase and glucose-6-phosphate-1-dehydrogenase were consistent in *Arabidopsis* in response to GSNO [35].

Flavones are naturally occurring metabolites with a protective biochemical function mostly identified in recent studies [36, 37]. It is well established that the protective effects of flavonoids against heavy metal stress are mainly attributed to three mechanisms, including clearing reactive oxygen species, chelating heavy metals and reducing DNA damage [38, 39]. Here the integrated data analysis of the T1 vs Cd compassion showed that the flavonoids isotrifoliin and acacetin were down-regulated. Similarly, the gene encoding flavonoid 3′, 5′-hydroxylase was down-regulated in T1 vs Cd, but not changed in T2 vs Cd. Conversely, the level of isotrifoliin was increased more than 4-fold in the T2 vs Cd comparison. There was a large difference in the content of isotrifoliin between the T1 and T2 treatment. It is speculated that isotrifoliin which functions as a non-enzymatic antioxidant competed with the enzymatic antioxidants, such as peroxidase and catalase, to quench ROS. The possible evidence might be the upregulation of most genes encoding enzymatic antioxidants like catalase in T1 vs Cd condition, or the downregulation of the gene (CYP75A) encoding flavonoid 3′, 5′-hydroxylase, which results in the decline of flavone biosynthesis. According to a similar study, the content of Cu and total phenolics as non-enzymatic antioxidants was promoted by SNP treatment but depleted by Cu and SNP simultaneous treatment [40]. Moreover, these results implied that there are various mechanisms through which NO-improved antioxidant capacity, including regulating the genes related to enzymatic antioxidants indirectly or taking advantage of nonenzymatic antioxidants to scavenge ROS and protect the cells from oxidative damage directly.

### Secondary metabolites to chelate and sequestrate cadmium

Under cadmium stress, the growth of plants was inhibited, and changes in the level of secondary metabolites were observed. This is one of the defense strategic mechanisms when the plant is subjected to heavy metal stress [41–43]. In addition, some studies have shown that Cd and Cu toxicity transformed certain substrates from primary metabolites to secondary defensive compounds, thereby inducing secondary metabolite production [44]. However, the NO donor SNP can affect secondary metabolite biosynthesis [45]. Furthermore, some secondary metabolites like phenylpropanoidand flavones are mostly mentioned for chelating metal ions, which is one of the proposed mechanisms of action in stressed plants [46, 47]. As shown in the phenylpropanoid biosynthesis pathway, the amount of transcript encoding peroxidase (POD) that was upstream of lignin was decreased, which implied that NO may influence the absorption of cadmium via lignin during long-term cadmium treatment. In addition, the phenylpropanoid pathway is responsible for the biosynthesis of lignin, flavonoids and benzenoids etc., which all play a role in protecting the plant against biotic and abiotic stress [48, 49]. Trans-cinnamic acid acts as a mediator during the catalytic process of phenylalanine to secondary metabolites such as lignin, which indicates its key function in the phenylpropanoid biosynthesis pathway [50]. In the present study, trans-cinnamic acid that acts as a precursor of many secondary substances was up-regulated in the T1 vs Cd comparison, but was down-regulated in the T2 vs Cd comparison. The finding suggested that the trans-cinnamic acid could alleviate cadmium stress in tall fescue. Studies have shown that the addition of trans-cinnamic acid to stressed plants had an inverse effect when compared to sole stress treatment [51, 52]. In feverfew plants, the exogenous trans-cinnamic acid decreased the absorption of Cu or Cd by inhibiting phenylalanine ammonia-lyase to reduce phenylpropanoids as metal chelators [53]. Furthermore, the trans-cinnamic acid levels also explained the difference in the cadmium content (Fig. 2). Moreover, the content of trans-cinnamic acid may affect the flavones, which are located downstream of the phenylpropanoid pathway. In the related analysis, we screened similar genes for phenylpropanoid biosynthesis and trans-cinnamic acid, most of which are part of CYP superfamily, such as CYP73A (trans-cinnamate 4-monooxygenase) and CYP84A (ferulate-5-hydroxylase). Trans-cinnamate 4-monooxygenase that is also called cinnamate 4-hydroxylase (C4H), participates as a member of the cytochrome P450 monooxygenases (P450s) in the synthesis of numerous polyphenoid compounds, such as flavonoids and lignin. In addition, the transcripts regulated the synthesis of the related secondary metabolites in the phenylpropanoid biosynthesis pathway like CCR (encoding cinnamoyl-CoA reductase) which showed a strong response to the T1 treatment, indicating that the phenylpropanoid biosynthesis pathway may play important roles in the defense mechanisms. Many studies have revealed similar significances [54, 55]. The study of the *CYP73A9v1* and *CYP82A1v2* genes has expounded on the complex molecular characterization of pea plant defense [56]. Moreover, the CYP71, 72, and 99 families have been found as temperature-responsive genes in perennial ryegrass and tall fescue [57]. In addition, SoCYP85A1was shown to enhance root development and drought stress tolerance in tomatoes [58].

Additionally, the role of flavones in clearing ROS has been discussed above, whereas previous research has indicated that flavonoids possessing the appropriate structural characteristics are efficient copper chelators [59]. Unfortunately, the metabolites and genes involved in the flavone and flavonol biosynthesis were down-regulated in our related analysis. Maybe, the fewer chelators like flavones led to the less content of Cd in the T1 vs Cd condition. A similar study found that cadmium content was decreased in the SNP treatment and increased in the c-PTIO treatment compared to the Cd treatment [32]. The results were consistent with our study in the absorption of Cd. Certainly, the reasons were varied. Perhaps the accumulation of flavonoids was dependent on the cadmium contaminant level and the period of cadmium exposure. Consequently, more detailed mechanisms must be researched in subsequent studies.

### The NO-regulated ABC transporter pathway is involved in regulating cadmium stress

With the application of exogenous NO, plants developed various mechanisms to defend against heavy metal stresses [60]. The ultimate goal of the adaptive mechanisms is to bind to the toxic ion by using phytochelatins and then sequester it in the vacuole [61]. Among the mechanisms, the regulation of the membrane transporter system is crucial during metal stress response [62]. Particularly, some metabolites highly activated ABC transporters in the present study. As shown in Table S6, carnitine and L-alanine, induced by exogenous NO, were up-regulated. In addition, findings in bacteria have shown that metabolites related to the ABC transporters could complex or chelate Cd^2+^ as low-molecular-weight organic acids, which might indicate their key roles in alleviating Cd stress [63]. In concordance with the metabolite results, the ABC transporters including ABCB1 and ABCC10 were largely accumulated in the roots of SNP treated tall fescue. Recently, the biological functions of ABCB transporters have been revealed in plants response to various stresses. The ClABCB, an auxin transporter gene, was up-regulated in the roots after NaCl treatment or PEG treatment [64]. Similarly, ABCC transporters have been explored in various plants, such as maize and wheat [65–68]. The above reports showed the valuable roles of ABC transporters and offered initiative for further characterizing their biological implications. Furthermore, TaABCC3, a prime candidate for plant genetic engineering to enhance tolerance, was induced substantially by GA3 [66]. Similar results were also observed in other studies (AtABCC13/ABCC11) [69]. ABCC genes play a key role in integrated pathways under multiple abiotic stresses, and the effects of some other signaling molecules (i.e. ABA and MeJA) on its transcript accumulation have been well investigated [70]. Thus, we speculate that NO, which is a multifunctional signaling molecule, plays a vital role in regulating the ABC transporters to improve Cd transport and detoxification.

## Conclusion

**Fig. 7:**
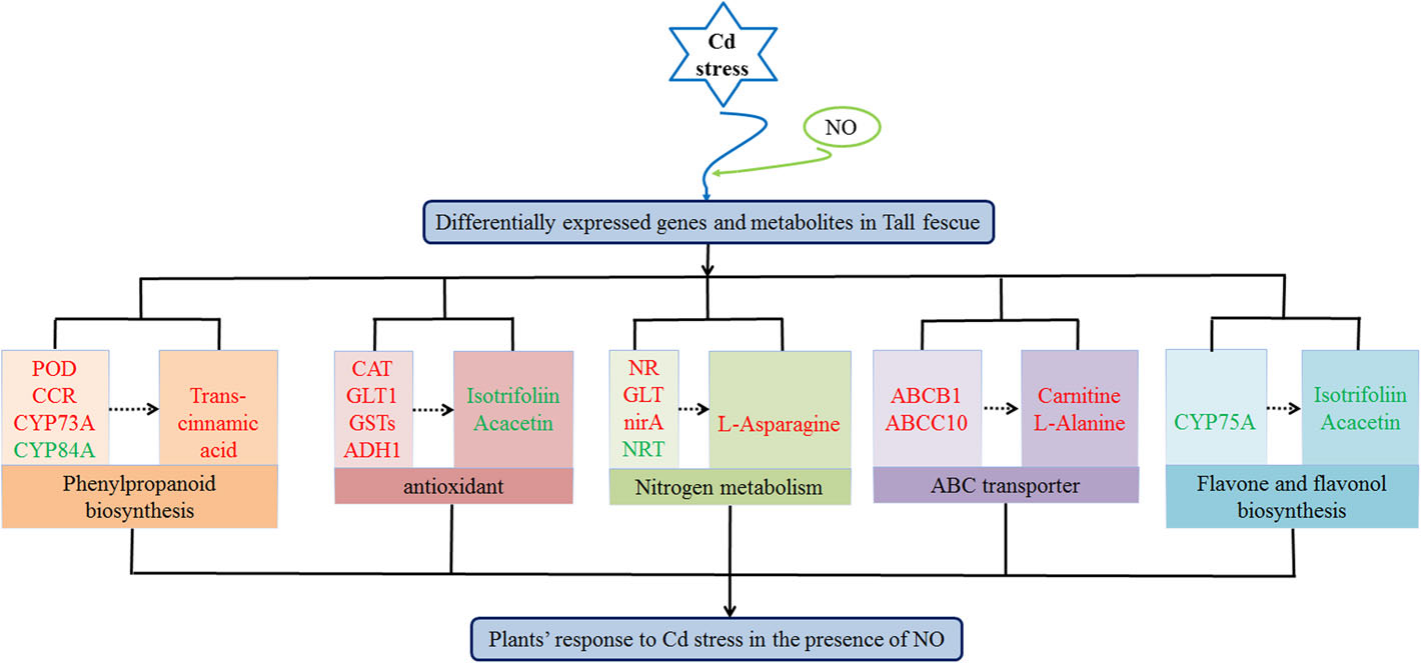
A proposed model of nitric oxide (NO)-regulated cadmium stress response in tall fescue

In this study, integrated analyses of metabolome and transcriptome provide a deeper understanding of the molecular mechanisms of Cd detoxification by NO in tall fescue (Fig. 7). NO could regulate the expression of genes and metabolites involved in nitrogen metabolism to protect tall fescue against Cd stress. In addition, NO application increased the expression of genes encoding CAT, GLT1, GSTs and ADH1, and decreased the contents of isotrifoliin and acacetin, indicating that NO participates in the regulation of the antioxidant system. Additionally, the up-regulated genes like ABCB1 and ABCC10 upon NO application highly activated the synthesis of ABC transporter related metabolites (carnitine and L-alanine), suggesting that NO plays a crucial role in regulating the ABC transporters pathway to improve Cd transportation and detoxification. Moreover, NO-regulated secondary metabolites were found and discussed in this study, especially the biosynthesis of phenylpropanoid, flavone and flavonol. Taken together, the NO application meliorate Cd stress induced injury through increasing antioxidant capacity, releasing more secondary metabolites for Cd chelation and sequestration, and regulating heavy metal transporters.

## Methods

### Plant materials and stress treatment

A commercial type tall fescue ‘houndog 5’ (Beijing Best Grass Industry Co., Ltd) was seeded in a plastic pot (7.5 cm in diameter and 9.0 cm deep) filled with pearlstone and vermiculite (1:1, v/v). After germination, the plants were kept in the greenhouse with a daily maximum/minimum temperature of 24/20 °C for a 16 h photoperiod (300 μmol photons m^− 2^ s^− 1^ PAR) for 30 days allowing the roots and shoots to establish [71]. The tall fescue seedlings were then moved to flasks and acclimated in 1/2 Hoagland solution for 2 weeks. The seedlings were then divided into eight treatment regimes including 1/2 Hoagland solution (Control), 1/2 Hoagland solution with 50 mg/L Cd^2+^ (CdCl_2_·2.5H_2_O) (Cd treatment), 1/2 Hoagland solution with 50 mg/L Cd^2+^ and 200 μM SNP (T1 treatment), and 1/2 Hoagland solution with 50 mg/L Cd^2+^, 200 μM L-NAME and 100 μM c-PTIO (T2 treatment), 200 μM L-NAME alone, 100 μM c-PTIO alone, 200 μM L-NAME together with 100 μM c-PTIO and 200 μM SNP alone. Each treatment was performed with three replications. SNP, L-NAME and c-PTIO were used as NO donor, NO production inhibitor and NO scavenger, respectively. After 48 h of treatment, the tall fescue seedlings were collected, and the roots and shoots were separated and washed with deionized water twice and blotted with tissue paper to dry. Afterwards, the root length was measured and the NO content in tall fescue root was detected using NO-specific cell-permeable fluorescent probe 3-amino, 4-aminomethyl-2,7-difluoro-fluorescein diacetate (DAF-FM DA, excitation at 488 nm, emission at 525 nm; Calbiochem, San Diego, CA, USA) as described [72]. The activities of superoxide dismutase (SOD) and ascorbate peroxidase (APX) were measured according to the method of our previous research [73, 74]. The samples were oven-dried at 105 °C for 15 min and then dried at 80 °C until a constant weight was attained and recorded as the dry weight. Subsequently, the Cd content was determined by the method described previously [74]. Additionally, the fresh root samples were frozen in liquid nitrogen and stored at − 80 °C until further analysis were performed.

### Transcriptome analysis

Total RNA was extracted from the roots of tall fescue using the Trizol reagent according to the manufacturer’s protocol (Invitrogen, USA). The purity and quality of total RNA were checked as described previously [75]. High quality total RNA samples were reverse transcribed into cDNA and used in cDNA library construction. Sequencing was performed on an Illumina Hiseq platform in Novogene. Based on the sequence information, gene functional annotation, differential expression analysis, GO and KEGG pathway enrichment analyses and quantitative RT-PCR analyses were performed as described previously [25].

### Metabolite profiling

Metabolite profiling was carried out using a widely targeted metabolome method at Wuhan Metware Biotechnology Co., Ltd. (Wuhan, China) (http://www.metware.cn/). The freeze-dried root was crushed using a mixer mill (MM 400, Retsch) with a zirconia bead for 1.5 min at 30 Hz under frozen conditions. Sample (100 mg) was extracted overnight at 4 °C with 1.0 mL 70% aqueous methanol, then adsorbed and filtrated before analysis using a LC–electrospray ionization (ESI)-MS/MS system. Quantification of metabolites was carried out using a multiple reaction monitoring (MRM) method as described previously [76].

### Statistical analysis

The statistical analysis was carried out by a one-way or two-way analysis of variance using SPSS (SPSS Inc., USA, version18.0), followed by comparisons of means using the least significant difference (LSD) multiple range test. Values are given as mean ± SE. Capital letters indicate the significant difference at *P* < 0.01. Small letters and Asterisks (*) indicate the significant difference at *P* < 0.05. Pearson’s correlation was carried out to confirm the reliability of the Illumina RNA-Seq. All the metabolomics data discussed below showed significant fold changes based on pairwise t testing at a significance level of *P* < 0.05.

## Supplementary information

**Additional file 1:** **Table S1.** Relative fluorescence intensity in tall fescue roots. The fluorescence was quantified with ImageJ program and registered in fifteen squares of 1000 μm^2^ each photo. Values are presented as a ration to the untreated Con (n = 4). **Table S2.** Significance at Cd level in Table 1 analyzed by LSD test. The significant difference was presented as capital letters at *P* < 0.01and small letters at *P* < 0.05, respectively. **Table S3.** Significance at NO level in Table 1 analyzed by LSD test. The significant difference was presented as capital letters at P < 0.01and small letters at P < 0.05, respectively. **Table S4.** Summary of sequence assembly after illumina sequencing. **Table S5.** Length distribution of the transcripts and unigenes clustered from the de novo assembly. **Table S6.** Different metabolite levels inT1 treatment and Cd treatment in tall fescue. **Table S7.** The fold changes of selected metabolitesin T1 vs Cd comparison. **Table S8.** The level of related DEGs and different metabolites response to T1 treatment vs Cd treatment. **Figure S1.** Volcano Plots and Venn diagrams of significantly differentially expressedtranscripts in the tall fescue roots with or without NO treatment under cadmium stress. (a) Volcanoplot in T1vsCd. (b) volcano plot in T2vsCd. (c) Venn diagram analysis in different treatment. (d) Venn diagram analysis between T1vsCd and T2vsCd. Numbers indicate the number of transcripts with significant changes inexpression under different conditions. Overlaps indicate the number of common transcripts differentially expressed, and numbers outside overlaps indicate the number of cultivar or subgroup specific transcripts differentially expressed. There were three regime, comprising Cd, T1, and T2. They respectively presented the tall fescue seedlings were cultivated in 1/2 Hoagland solution with 50 mg/L Cd^2+^ (CdCl_2_•2.5H_2_O) (Cd treatment), 1/2 Hoagland solution with 50 mg/L Cd^2+^ and 200 μM SNP (T1 treatment) and 1/2 Hoagland solution with 50 mg/L Cd^2+^, 200 μM L-NAME and 100 μM c-PTIO (T2 treatment). Each value is the mean of three replicates. **Figure S2.** Histogram of the gene ontology classification analysis of the DEGs in response to theT1 treatment in tall fescue roots. The tall fescue seedlings were cultivated in 1/2 Hoagland solution with 50 mg/L Cd^2+^ (CdCl_2_•2.5H_2_O) (Cd treatment) and 1/2 Hoagland solution with 50 mg/L Cd^2+^ and 200 μM SNP (T1 treatment), respectively. Each value is the mean of three replicates. **Figure S3.** The top 10 metabolites according to the VIP values in tall fescue under T1 treatment. The tall fescue seedlings were cultivated in 1/2 Hoagland solution with 50 mg/L Cd^2+^ (CdCl_2_•2.5H_2_O) (Cd treatment) and 1/2 Hoagland solution with 50 mg/L Cd^2+^ and 200 μM SNP (T1 treatment), respectively. Each value is the mean of three replicates. **Figure S4.** Principal component analysis (PCA) of the metabolite profiles in tall fescue roots. The analysis was performed on all the metabolites detected in tall fescue roots under different conditions. There were four treatment regimes in this study, including the Con, Cd treatment, T1 treatment, and T2 treatment, and each regime had three replicates. They respectively presented the tall fescue seedlings were cultivated in 1/2 Hoagland solution (Con), 1/2 Hoagland solution with 50 mg/L Cd^2+^ (CdCl_2_•2.5H_2_O) (Cd treatment), 1/2 Hoagland solution with 50 mg/L Cd^2+^ and 200 μM SNP (T1 treatment), and 1/2 Hoagland solution with 50 mg/L Cd^2+^, 200 μM L-NAME and 100 μM c-PTIO (T2 treatment). **Figure S5.** The hierarchical cluster analysis (HCA) of the differentially expressed metabolites selected from the integrated analysis between the T1 treatment and Cd treatment in tall fescue roots. The tall fescue seedlings were cultivated in 1/2 Hoagland solution with 50 mg/L Cd^2+^ (CdCl_2_•2.5H_2_O) (Cd treatment) and 1/2 Hoagland solution with 50 mg/L Cd^2+^ and 200 μM SNP (T1 treatment), respectively. Each value is the mean of three replicates. **Figure S6.** The distribution of metabolites in different KEGG pathways. (A) The metabolites in Cd vs Con. (B) the metabolites in T1 vs Cd. There were three regime, comprising Cd, T1 and T2. They respectively presented the tall fescue seedlings were cultivated in 1/2 Hoagland solution with 50 mg/L Cd^2+^ (CdCl_2_•2.5H_2_O) (Cd treatment), 1/2 Hoagland solution with 50 mg/L Cd^2+^ and 200 μM SNP (T1 treatment) and 1/2 Hoagland solution with 50 mg/L Cd^2+^ and 200 μM NG-nitro-L-Arg-methyl ester (L-NAME) and 100 μM 2-(4-carboxyphenyl)-4,4,5,5-tetramethylimidazoline-1-oxyl-3-oxide (cPTIO) (T2 treatment). **Figure S7.** The activities of SOD (superoxide dismutase) and APX (Ascorbate peroxidase) in tall fescue roots. Values were given as means ± SD (*n* = 4). Data about Cd, T1 and T2 treatment were analyzed using one-way Analysis of Variance, followed by LSD test. Asterisks (*) indicate the significant difference at *P* < 0.05. **Figure S8.** Correlations of expression level analyzed by RNA-Seq platform (y axis) with data resulted from qRT-PCR (x axis).

## Data Availability

The datasets generated and analyzed during the current study are available in the NCBI Sequence Read Archive repository under project number PRJNA648793 (https://www.ncbi.nlm.nih.gov/Traces/study/?acc=PRJNA648793). The Supplementary Material (Additional file) for this article can be found online.

## Abbreviations

Cd: Cadmium
NO: nitric oxide
SNP: Sodium nitroprusside
c-PTIO: 2-phenyl-4, 4, 5, 5-tetramethylimidazoline-1-oxyl-3-oxide
DEG: Differentially expressed genes
