# Supplementary material for: Comparative transcriptome combined with metabolome analyses revealed key factors involved in nitric oxide (NO)-regulated cadmium stress adaptation in tall fescue
Source: BMC Genomics. 2020 Aug 31;21:601. doi: 10.1186/s12864-020-07017-8 (PMC7457814; doi:10.1186/s12864-020-07017-8)
Supplement: Supplementary file 1 — Additional file 1: Table S1. Relative fluorescence intensity in tall fescue roots. The fluorescence was quantified with ImageJ program and registered in fifteen squares of 1000 μm2 each photo. Values are presented as a ration to the untreated Con (n = 4). Table S2. Significance at Cd level in Table 1 analyzed by LSD test. The significant difference was presented as capital letters at P < 0.01and small letters at P < 0.05, respectively. Table S3. Significance at NO level in Table 1 analyzed by LSD test. The significant difference was presented as capital letters at P < 0.01and small letters at P < 0.05, respectively. Table S4. Summary of sequence assembly after illumina sequencing. Table S5. Length distribution of the transcripts and unigenes clustered from the de novo assembly. Table S6. Different metabolite levels inT1 treatment and Cd treatment in tall fescue. Table S7. The fold changes of selected metabolitesin T1 vs Cd comparison. Table S8. The level of related DEGs and different metabolites response to T1 treatment vs Cd treatment. Figure S1. Volcano Plots and Venn diagrams of significantly differentially expressedtranscripts in the tall fescue roots with or without NO treatment under cadmium stress. (a) Volcanoplot in T1vsCd. (b) volcano plot in T2vsCd. (c) Venn diagram analysis in different treatment. (d) Venn diagram analysis between T1vsCd and T2vsCd. Numbers indicate the number of transcripts with significant changes inexpression under different conditions. Overlaps indicate the number of common transcripts differentially expressed, and numbers outside overlaps indicate the number of cultivar or subgroup specific transcripts differentially expressed. There were three regime, comprising Cd, T1, and T2. They respectively presented the tall fescue seedlings were cultivated in 1/2 Hoagland solution with 50 mg/L Cd2+ (CdCl2•2.5H2O) (Cd treatment), 1/2 Hoagland solution with 50 mg/L Cd2+ and 200 μM SNP (T1 treatment) and 1/2 Hoagland solution with 50 mg/L Cd2+, [file 12864_2020_7017_MOESM1_ESM.docx]

**Supplemental material**

**Table S1.** Relative fluorescence intensity in tall fescue roots. The fluorescence was quantified with ImageJ program and registered in fifteen squares of 1000 μm^2^ each photo. Values are presented as a ration to the untreated Con (n = 4).

| Treatment | SNP | c-PTIO | L-NAME | L-NAME+c-PTIO | Con | Cd | T1 | T2 |
| --- | --- | --- | --- | --- | --- | --- | --- | --- |
| NO content | 1.97 | 0.83 | 0.71 | 0.28 | 1.00 | 1.38 | 2.44 | 0.92 |

**Table S2.** Significance at Cd level in Table 1 analyzed by LSD test. The significant difference was presented as capital letters at P < 0.01and small letters at P < 0.05, respectively.

| Cd | Mean Xi | Significance | |
| --- | --- | --- | --- |
|  |  | α=0.05 | α=0.01 |
| With Cd | 1.58 | a | A |
| Without Cd | 1.08 | b | B |

**Table S3.** Significance at NO level in Table 1 analyzed by LSD test. The significant difference was presented as capital letters at P < 0.01and small letters at P < 0.05, respectively.

| NO | Mean Xi | Significance | |
| --- | --- | --- | --- |
|  |  | α=0.05 | α=0.01 |
| SNP | 1.58 | a | A |
| Without NO addition^†^ | 1.08 | b | B |
| L-NAME+c-PTIO | 0.60 | c | C |

^†^ “Without NO addition” refers to Control and Cd treatment

**Table S4.** Summary of sequence assembly after illumina sequencing.

| Sample**^†^** | Raw Reads | Clean Reads |  | Clean Bases | Error (%) | Q20 (%) | Q30 (%) | GC (%) |
| --- | --- | --- | --- | --- | --- | --- | --- | --- |
| Con1 | 62584984 | 60907468 |  | 9.14G | 0.01 | 97.59 | 93.81 | 53.7 |
| Con2 | 46259288 | 45480844 |  | 6.82G | 0.01 | 97.32 | 93.16 | 50.37 |
| Con3 | 53117390 | 51810710 |  | 7.77G | 0.01 | 97.44 | 93.54 | 52.08 |
| Cd1 | 58625108 | 57334986 |  | 8.6G | 0.01 | 97.29 | 93.06 | 55.42 |
| Cd2 | 49789754 | 48859078 |  | 7.33G | 0.02 | 97.06 | 92.62 | 55.23 |
| Cd3 | 52984272 | 51976104 |  | 7.8G | 0.02 | 97.11 | 92.69 | 55.36 |
| T11 | 48057290 | 47193350 |  | 7.08G | 0.02 | 97.13 | 92.76 | 55.11 |
| T12 | 54031232 | 52886932 |  | 7.93G | 0.01 | 97.42 | 93.32 | 55.03 |
| T13 | 50177600 | 49288134 |  | 7.39G | 0.01 | 97.32 | 93.14 | 55.14 |
| T21 | 55495822 | 54533056 |  | 8.18G | 0.01 | 97.22 | 92.92 | 55.48 |
| T22 | 46965256 | 46078954 |  | 6.91G | 0.01 | 97.43 | 93.3 | 55.55 |
| T23 | 59562528 | 58495636 |  | 8.77G | 0.01 | 97.18 | 92.82 | 55.42 |

**^†^** The sample were from four treatment regimes in this study, including the control (Con), Cd treatment, T1 treatment, and T2 treatment, and each regime had three replicates. They respectively presented the tall fescue seedlings were cultivated in 1/2 Hoagland solution (control, Con), 1/2 Hoagland solution with 50 mg/L Cd^2+^ (CdCl_2_•2.5H_2_O) (Cd treatment), 1/2 Hoagland solution with 50 mg/L Cd^2+^ and 200 μM SNP (T1 treatment), and 1/2 Hoagland solution with 50 mg/L Cd^2+^, 200 μM L-NAME and 100 μM c-PTIO (T2 treatment).

**Table S5** Length distribution of the transcripts and unigenes clustered from the de novo assembly.

| Category | Transcripts | Unigenes |
| --- | --- | --- |
| 200-500bp | 1317122 | 705398 |
| 500-1kbp | 472146 | 183636 |
| 1k-2kbp | 181139 | 66912 |
| >2kbp | 35170 | 12978 |
| Total | 2005577 | 968924 |
| N50 | 654 | 560 |
| N90 | 258 | 241 |
| max length | 14405 | 14405 |
| min length | 201 | 201 |
| mean length | 534 | 475 |

**Table S6.** Different metabolite levels in T1 treatment and Cd treatment in tall fescue. The tall fescue seedlings were cultivated in 1/2 Hoagland solution with 50 mg/L Cd^2+^ (CdCl_2_•2.5H_2_O) (Cd treatment) and 1/2 Hoagland solution with 50 mg/L Cd^2+^ and 200 μM SNP (T1 treatment), respectively. Each value is the mean of three replicates.

| Compounds | Class | Fold.Change | type |
| --- | --- | --- | --- |
| L-Alanine | Amino acids | 2.537444934 | up |
| L-Citrulline | Amino acids | 2.362606232 | up |
| L-PyroglutamicAcid  L-Asparagine | Amino acids  Amino acids | 4.923076923  2.12 | up  up |
| L-Asparagine Anhydrous | Amino acids | 2.357769974 | up |
| Homocystine | Amino acids | 0.390628707 | down |
| (-)-3-(3,4-Dihydroxyphenyl)-2-methyllane | Amino acid derivatives | 2.008572149 | up |
| 1-Methylhistidine | Amino acid derivatives | 3.295519002 | up |
| D-Pipecolinic acid | Amino acid derivatives | 2.006944444 | up |
| L-Homocitrulline | Amino acid derivatives | 2.06190823 | up |
| N6-Acetyl-L-lysine | Amino acid derivatives | 2.049333333 | up |
| Lysine butyrate | Amino acid derivatives | 2.68695979 | up |
| Kinurenine | Amino acid derivatives | 2.144266338 | up |
| Quisqualic acid | Aminoacid derivatives | 3.601286174 | up |
| sn-Glycero-3-phosphocholine | Cholines | 2.086795937 | up |
| Coumaroyl choline | Cholines | 0.423382046 | down |
| epicatechingallate(ECG) | Catechin and its derivatives | 2.489944212 | up |
| (-)-Epicatechingallate | Catechin and its derivatives | 2.342157594 | up |
| (-)-catechingallate | Catechin and its derivatives | 2.862821789 | up |
| catechingallate(CG) | Catechin and its derivatives | 2.152084102 | up |
| N',N",N"'-p-coumaroyl-cinnamoyl-caffeoylspermidine | Phenolamides | 0.378830409 | down |
| N-Feruloylspermidine | Phenolamides | 0.453575482 | down |
| N-p-coumaroylagmatine | Phenolamides | 0.397212544 | down |
| N-feruloylagmatine | Phenolamides | 0.389864526 | down |
| N-p-coumaroylagmatine iso2  Putrescine derivative | Phenolamides  Phenolamides | 0.369649805  2.050096805 | down  up |
| N-Caffeoylagmatine | Phenolamides | 0.197477787 | down |
| 2'-Deoxycytidine-5'-diphosphate | Nucleotide and its derivates | 2.731804586 | up |
| 2'-Deoxyguanosine | Nucleotide and its derivates | 0.404396371 | down |
| 6-Benzylaminopurine | Nucleotide and its derivates | 2.539940828 | up |
| 8-Hydroxyguanosine | Nucleotide and its derivates | 0.494842233 | down |
| Uracil | Nucleotide and its derivates | 0.490692707 | down |
| Delphinidin 3-Glu | Anthocyanin | 2.996270203 | up |
| Malvidin 3-O-galactoside | Anthocyanin | 2.573048408 | up |
| Peonidin | Anthocyanin | 360.7037037 | up |
| Peonidin O-hexoside | Anthocyanin | 10.258972 | up |
| Cyanidin O-syringic acid | Anthocyanin | 4.939682423 | up |
| Cyanidin O-hexosyl-O-hexosyl-O-hexoside | Anthocyanin | 3.147249191 | up |
| KUROMANIN CHLORIDE | Anthocyanin | 6.103195995 | up |
| Cyanidin 3-galactoside (Idaein chloride) | Anthocyanin | 5.868323816 | up |
| Cyanidin 3-glucoside | Anthocyanin | 6.109303839 | up |
| Cyanidin 3-rutinoside (Keracyanin) | Anthocyanin | 4.205819156 | up |
| Rosinidin 3-O-glucoside | Anthocyanin | 2.207259953 | up |
| 3,7-Dihydroxy-3',4'-dimethoxyflavone | Flavone | 0.467307692 | down |
| Acacetin | Flavone | 0.000417195 | down |
| Chrysoeriol 7-O-hexoside | Flavone | 3.077022112 | up |
| Chrysoeriol 7-O-rutinoside  Chrysoeriol O-malonylhexoside | Flavone  Flavone | 3.699262546  3.013590703 | up  up |
| Chrysoeriol O-hexoside | Flavone | 2.255828319 | up |
| Chrysoeriol O-rhamnosyl-O-hexoside | Flavone | 5.205128205 | up |
| Chrysoeriol 5-O-hexoside  Tricin 5-O-hexoside | Flavone  Flavone | 3.685714286  2.292136164 | up  up |
| Tricin 5-O-hexosyl-O-hexoside | Flavone | 5.016227444 | up |
| Tricin 7-O-hexoside | Flavone | 2.390762289 | up |
| Luteolin O-rutinoside | Flavone | 0.099524946 | down |
| Apigenin O-malonylhexoside | Flavone | 5.238995693 | up |
| Avicularin | Flavone | 0.007969303 | down |
| Rhoifolin | Flavone | 99.92592593 | up |
| Rhoifolin (Apigenin7-O-neohesperidoside) | Flavone | 0.075325981 | down |
| Isorhoifolin | Flavone | 0.118077297 | down |
| sakuranetin | Flavone | 0.000318329 | down |
| C-hexosyl-apigenin O-hexoside | Flavonoids-C | 0.111496113 | down |
| C-hexosyl-apigenin O-p-coumaroylhexoside | Flavonoids-C | 0.001139241 | down |
| Vitexin 2''-O-beta-L-rhamnoside | Flavonoids-C | 2.51641791 | up |
| Quercetin-3-(6''-malonyl)-Glucoside | Flavonol | 0.393769866 | down |
| Kaempferol 3-O-robinoside (Biorobin) | Flavonol | 0.108374172 | down |
| Kaempferol-3-Glucoside-6''-p-coumaroyl | Flavonol | 0.180242311 | down |
| Kaempferitrin (Kaempferol 3,7-dirhamnoside) | Flavonol | 0.181373744 | down |
| 1-O-caffeoylquinic acid | Quinate and its derivatives | 3.201612903 | up |
| 4'-Hydroxy-5,7-dimethoxyflavanone | Flavonoids | 2.593309859 | up |
| 3,4',5,7-tetrahydroxy-3'-methoxyflavone | Flavonoids | 2.01627907 | up |
| 2,5-Dihydroxybenzoate | Others | 0.491635688 | down |
| 2,6-Xylidine | Others | 2.017184943 | up |
| 2-Amino-1,3-octadecanediol | Others | 2.822037878 | up |
| 8-Chlro-1-tetrahydronorharmanone | Others | 2.094071806 | up |
| alpha-(3,4-dihydroxyphenyl)lactic acid | Others | 3.660508083 | up |
| Phloridzin  Xanthoxylin | Others  Others | 3.089959373  0.360176391 | up  down |
| Phellodenol H | Others | 2.808445129 | up |
| 12OHJA-Ile-1 | Others | 2.297953356 | up |
| Carnitine | Others | 2.744807122 | up |
| Griseofulvin | Others2 | 0.491253644 | down |
| sesamin | Others2 | 2.234042553 | up |
| 1-O-b-D-glucopyranosyl sinapate | Hydroxycinnamoyl derivatives | 0.255670103 | down |
| Disinapoylhexoside | Hydroxycinnamoyl derivatives | 2.663120567 | up |
| trans-Cinnamic acid | Hydroxycinnamoyl derivatives | 2.705971277 | up |
| Pipecolate | Alkaloids | 2.346207865 | up |
| 2-Propenyl (sinigrin) | Carbohydrates | 0.253641618 | down |
| D(-)-Threose | Carbohydrates | 0.322994652 | down |
| Ribitol | Carbohydrates | 3.53649635 | up |
| Cafestol  Oryzalexin?C | Terpenoids  Terpenoids | 2.039835339  2.191019648 | up  up |
| 6-hydroxynicotinic acid | Nicotinic acid and its derivatives | 0.294462901 | down |
| 2-Picolinic acid | Organic acid and its derivatives | 0.49 | down |
| 2–furanoic acid | Organic acid and its derivatives | 2.131595282 | up |
| DL-Pipecolinic acid | Organic acid and its derivatives | 2.163321799 | up |
| Quisqualate | Organic acid and its derivatives | 0.461415306 | down |
| 13-HPODE | Lipids-fatty acid | 0.393536122 | down |
| 3-Indolebutyric acid | Phytohormones | 4.078031809 | up |

**Table S7** The fold changes of selected metabolites in T1 vs Cd comparison**^†^**

| Compounds | Class | Fold. Change**^†^** |
| --- | --- | --- |
| Peonidin | Anthocyanin | 360.7037 |
| Rhoifolin | Flavone | 99.92593 |
| Peonidin O-hexoside | Anthocyanin | 10.25897 |
| Cyanidin 3-glucoside | Anthocyanin | 6.109304 |
| KUROMANIN CHLORIDE | Anthocyanin | 6.103196 |
| Avicularin | Flavone | 0.007969 |
| C-hexosyl-apigenin O-p-coumaroylhexoside | Flavonoids-C | 0.001139 |
| Acacetin | Flavone | 0.000417 |
| Sakuranetin | Flavone | 0.000318 |

**^†^**The tall fescue seedlings were cultivated in 1/2 Hoagland solution with 50 mg/L Cd^2+^ (CdCl_2_•2.5H_2_O) (Cd treatment) and 1/2 Hoagland solution with 50 mg/L Cd^2+^ and 200 μM SNP (T1 treatment), respectively. Each value is the mean of three replicates. Refer to the ratio of the concentration of metabolites of T1 treatment to the concentration of metabolites of Cd treatment.

**Table S8.** The level of related DEGs and different metabolites response to T1 treatment vs Cd treatment. The tall fescue seedlings were cultivated in 1/2 Hoagland solution with 50 mg/L Cd^2+^ (CdCl_2_•2.5H_2_O) (Cd treatment) and 1/2 Hoagland solution with 50 mg/L Cd^2+^ and 200 μM SNP (T1 treatment), respectively. Each value is the mean of three replicates.

| Term | Compounds | Class | Fold.Change(SNPvsCd) | Fold.Change(PLvsCd) | Gene_id | Description | log2ratio(SNPvsCd) | log2ratio(PLvsCd) |
| --- | --- | --- | --- | --- | --- | --- | --- | --- |
| ABC transporters | Carnitine | Others | 2.7448 | 0.5292 | TRINITY_DN400262_c7_g2 | ATP-binding cassette, subfamily B (MDR/TAP), member 1 | 1.5112 | 0.60218 |
|  | L-Alanine | Amino acids | 2.5374 | 1.4626 | TRINITY_DN370337_c1_g4 | ATP-binding cassette, subfamily B (MDR/TAP), member 1 | 1.9914 | 1.2374 |
|  |  |  |  |  | TRINITY_DN367086_c1_g2 | ATP-binding cassette, subfamily C (CFTR/MRP), member 10 | 2.8355 | 2.1535 |
| Phenylpropanoid biosynthesis | trans-Cinnamic acid | Hydroxycinnamoyl derivatives | 2.7060 | 1.4187 | TRINITY_DN384579_c0_g2 | peroxidase | -1.833 | -0.8813 |
|  |  |  |  |  | TRINITY_DN338664_c3_g1 | peroxidase | -2.1992 | -0.7401 |
|  |  |  |  |  | TRINITY_DN367754_c1_g1 | trans-cinnamate 4-monooxygenase | 2.5086 | 2.0206 |
|  |  |  |  |  | TRINITY_DN345973_c0_g1 | cinnamoyl-CoA reductase | 3.4356 | 1.2925 |
|  |  |  |  |  | TRINITY_DN386671_c2_g5 | peroxidase | -1.8802 | -0.2061 |
|  |  |  |  |  | TRINITY_DN359843_c1_g1 | trans-cinnamate 4-monooxygenase | 2.7516 | 1.9301 |
|  |  |  |  |  | TRINITY_DN400412_c2_g2 | ferulate-5-hydroxylase | -1.3505 | -0.4690 |
|  |  |  |  |  | TRINITY_DN359843_c2_g1 | trans-cinnamate 4-monooxygenase | 2.3034 | 1.7221 |
|  |  |  |  |  | TRINITY_DN380055_c1_g4 | trans-cinnamate 4-monooxygenase | 2.8636 | 2.0238 |
|  |  |  |  |  | TRINITY_DN367872_c0_g4 | trans-cinnamate 4-monooxygenase | 2.286 | 1.7238 |
|  |  |  |  |  | TRINITY_DN351829_c0_g4 | peroxidase | -1.8577 | -0.1923 |
|  |  |  |  |  | TRINITY_DN380055_c1_g2 | trans-cinnamate 4-monooxygenase | 2.4327 | 1.8606 |
| Phenylalanine metabolism | trans-Cinnamic acid | Hydroxycinnamoyl derivatives | 2.7060 | 1.4187 | TRINITY_DN359843_c1_g1 | trans-cinnamate 4-monooxygenase | 2.7516 | 1.9301 |
|  |  |  |  |  | TRINITY_DN359843_c2_g1 | trans-cinnamate 4-monooxygenase | 2.3034 | 1.7221 |
|  |  |  |  |  | TRINITY_DN380055_c1_g4 | trans-cinnamate 4-monooxygenase | 2.8636 | 2.0238 |
|  |  |  |  |  | TRINITY_DN367872_c0_g4 | trans-cinnamate 4-monooxygenase | 2.286 | 1.7238 |
|  |  |  |  |  | TRINITY_DN367754_c1_g1 | trans-cinnamate 4-monooxygenase | 2.5086 | 2.0206 |
|  |  |  |  |  | TRINITY_DN380055_c1_g2 | trans-cinnamate 4-monooxygenase | 2.4327 | 1.8606 |
| Flavone and flavonol biosynthesis | Isotrifoliin | Flavone | 0.1181 | 4.5783 | TRINITY_DN386812_c0_g3 | flavonoid 3',5'-hydroxylase | -1.7957 | -0.5861 |
|  | Acacetin | Flavone | 0.0004 | 0.3997 |  |  |  |  |
| Tyrosine metabolism | 2,5-Dihydroxybenzoate/2,5-dihydroxybenzoic acid | Organic acid and its derivatives | 0.4916 | 1.5613 | TRINITY_DN330049_c1_g1 | alcohol dehydrogenase class-P | 3.1165 | 0.4230 |
|  |  |  |  |  | TRINITY_DN351047_c0_g3 | alcohol dehydrogenase class-P | 3.8692 | 2.3934 |
|  |  |  |  |  | TRINITY_DN351303_c1_g2 | alcohol dehydrogenase class-P | 2.6383 | 1.5694 |
|  |  |  |  |  | TRINITY_DN336653_c1_g1 | alcohol dehydrogenase class-P | 2.2926 | 1.0464 |
|  |  |  |  |  | TRINITY_DN327493_c0_g1 | alcohol dehydrogenase class-P | 2.1846 | 1.3623 |
|  |  |  |  |  | TRINITY_DN346637_c0_g2 | alcohol dehydrogenase class-P | 2.1097 | 1.8310 |
|  |  |  |  |  | TRINITY_DN346637_c0_g1 | alcohol dehydrogenase class-P | 2.4206 | 1.5578 |
|  |  |  |  |  | TRINITY_DN330791_c2_g1 | alcohol dehydrogenase class-P | 2.9909 | 0.4736 |
|  |  |  |  |  | TRINITY_DN327493_c0_g2 | alcohol dehydrogenase class-P | 2.117 | 1.4849 |
|  |  |  |  |  | TRINITY_DN332185_c0_g2 | alcohol dehydrogenase class-P | 2.2426 | 1.0971 |
|  |  |  |  |  | TRINITY_DN330791_c2_g2 | alcohol dehydrogenase class-P | 2.0264 | 1.3224 |
|  |  |  |  |  | TRINITY_DN330791_c2_g6 | alcohol dehydrogenase class-P | 2.684 | 1.4922 |
|  |  |  |  |  | TRINITY_DN399931_c4_g2 | alcohol dehydrogenase class-P | 2.0172 | 1.2775 |
|  |  |  |  |  | TRINITY_DN336653_c1_g2 | alcohol dehydrogenase class-P | 2.2498 | 1.2887 |
|  |  |  |  |  | TRINITY_DN379442_c0_g2 | alcohol dehydrogenase class-P | 1.7648 | 1.7195 |
| Nitrogen metabolism | L-Asparagine | Amino acids | 2.1200 | 1.0940 | TRINITY_DN380554_c0_g2 | nitrate reductase (NAD(P)H) | 2.6907 | 0.0345 |
|  |  |  |  |  | TRINITY_DN380554_c0_g1 | nitrate reductase (NAD(P)H) | 2.5907 | 0.3174 |
|  |  |  |  |  | TRINITY_DN348303_c1_g2 | MFS transporter, NNP family, nitrate/nitrite transporter | -1.5092 | -0.3867 |
|  |  |  |  |  | TRINITY_DN348032_c3_g1 | nitrate reductase (NAD(P)H) | 1.8839 | -0.3130 |
|  |  |  |  |  | TRINITY_DN353219_c1_g1 | MFS transporter, NNP family, nitrate/nitrite transporter | -1.5166 | -0.4028 |
|  |  |  |  |  | TRINITY_DN338027_c1_g1 | glutamate synthase (NADPH/NADH) | 1.9273 | 1.2264 |
|  |  |  |  |  | TRINITY_DN396249_c7_g2 | ferredoxin-nitrite reductase | 1.4373 | 0.8337 |
|  |  |  |  |  | TRINITY_DN367739_c0_g2 | nitrate reductase (NAD(P)H) | 2.2001 | -0.0186 |
| Arginine and proline metabolism | L-Citrulline | Amino acids | 2.3626 | 1.6686 | TRINITY_DN346407_c0_g2 | ornithine decarboxylase | -3.8219 | -0.2918 |
|  |  |  |  |  | TRINITY_DN346407_c0_g3 | ornithine decarboxylase | -3.6495 | -0.2479 |
|  |  |  |  |  | TRINITY_DN325236_c8_g1 | arginase | -2.5241 | -1.0993 |
|  |  |  |  |  | TRINITY_DN334409_c0_g1 | ornithine decarboxylase | -3.0831 | -0.9593 |
|  |  |  |  |  | TRINITY_DN384769_c1_g2 | prolyl 4-hydroxylase | 2.0199 | 1.8756 |
|  |  |  |  |  | TRINITY_DN384769_c1_g1 | prolyl 4-hydroxylase | 2.0408 | 1.8226 |
| Glutathione metabolism | L-Pyroglutamic acid | Amino acids | 4.9231 | 0.7554 | TRINITY_DN346407_c0_g2 | ornithine decarboxylase | -3.8219 | -0.2918 |
|  |  |  |  |  | TRINITY_DN346407_c0_g3 | ornithine decarboxylase | -3.6495 | -0.2479 |
|  |  |  |  |  | TRINITY_DN314196_c1_g1 | glutathione S-transferase | Inf | NA |
|  |  |  |  |  | TRINITY_DN334409_c0_g1 | ornithine decarboxylase | -3.0831 | -0.9593 |
|  |  |  |  |  | TRINITY_DN310547_c0_g1 | glucose-6-phosphate 1-dehydrogenase | -4.4403 | -2.7866 |
|  |  |  |  |  | TRINITY_DN384370_c2_g1 | glutathione S-transferase | 3.8466 | 0.3196 |
|  |  |  |  |  | TRINITY_DN358027_c0_g3 | glutathione S-transferase | 1.4609 | 0.8131 |
|  |  |  |  |  | TRINITY_DN348788_c0_g1 | glutathione S-transferase | 1.7319 | 2.0212 |
| Alanine, aspartate and glutamate metabolism | L-Asparagine | Amino acids | 2.1200 | 1.0940 | TRINITY_DN339215_c0_g1 | glutamate decarboxylase | 2.2886 | 0.3310 |
|  | L-Alanine | Amino acids | 2.5374 | 1.4626 | TRINITY_DN399803_c3_g7 | glutamate decarboxylase | 2.3775 | 0.3353 |
|  |  |  |  |  | TRINITY_DN353982_c0_g1 | glutamate decarboxylase | 2.4279 | 0.6460 |
|  |  |  |  |  | TRINITY_DN338027_c1_g1 | glutamate synthase (NADPH/NADH) | 1.9273 | 1.2264 |
| Taurine and hypotaurine metabolism | L-Alanine | Amino acids | 2.5374 | 1.4626 | TRINITY_DN339215_c0_g1 | glutamate decarboxylase | 2.2886 | 0.3310 |
|  |  |  |  |  | TRINITY_DN399803_c3_g7 | glutamate decarboxylase | 2.3775 | 0.3353 |
|  |  |  |  |  | TRINITY_DN353982_c0_g1 | glutamate decarboxylase | 2.4279 | 0.6460 |
|  |  |  |  |  | TRINITY_DN400526_c2_g1 | cysteamine dioxygenase | 1.6843 | 0.8041 |
| Carbon fixation in photosynthetic organisms | L-Alanine | Amino acids | 2.5374 | 1.4626 | TRINITY_DN375458_c1_g1 | malate dehydrogenase (NADP+) | -1.7817 | -0.8755 |
|  |  |  |  |  | TRINITY_DN385846_c1_g3 | triosephosphate isomerase (TIM) | 3.3106 | 1.9065 |
|  |  |  |  |  | TRINITY_DN400305_c8_g2 | pyruvate, orthophosphate dikinase | 1.9903 | 1.0770 |
|  |  |  |  |  | TRINITY_DN272308_c0_g1 | glyceraldehyde 3-phosphate dehydrogenase | -3.0477 | -0.8655 |
|  |  |  |  |  | TRINITY_DN385846_c1_g4 | triosephosphate isomerase (TIM) | 2.448 | 1.6071 |
| Cysteine and methionine metabolism | Homocystine | Amino acids | 0.3906 | 0.3550 | TRINITY_DN383055_c1_g1 | aspartate kinase | 1.8368 | 1.0351 |
|  | L-Alanine | Amino acids | 2.5374 | 1.4626 | TRINITY_DN367731_c3_g1 | L-3-cyanoalanine synthase/ cysteine synthase | 2.6683 | -0.1042 |
|  |  |  |  |  | TRINITY_DN383524_c1_g2 | aspartate kinase | 1.7086 | 1.0375 |
|  |  |  |  |  | TRINITY_DN366863_c0_g2 | DNA (cytosine-5)-methyltransferase 1 | 4.1621 | 2.9726 |
|  |  |  |  |  | TRINITY_DN365736_c2_g11 | S-adenosylmethionine synthetase | -2.4543 | -1.0510 |
|  |  |  |  |  | TRINITY_DN339974_c0_g1 | serine O-acetyltransferase | 0.01597 | 1.0000 |
|  |  |  |  |  | TRINITY_DN319764_c1_g1 | cystathionine beta-synthase | -2.9947 | -1.0426 |
|  |  |  |  |  | TRINITY_DN385145_c0_g2 | L-3-cyanoalanine synthase/ cysteine synthase | 3.6006 | 0.7211 |
|  |  |  |  |  | TRINITY_DN352327_c0_g1 | L-3-cyanoalanine synthase/ cysteine synthase | 2.183 | -0.4828 |
|  |  |  |  |  | TRINITY_DN319404_c6_g2 | adenosylhomocysteinase | -2.581 | -0.9434 |
|  |  |  |  |  | TRINITY_DN316068_c0_g1 | L-3-cyanoalanine synthase/ cysteine synthase | 2.5065 | -0.0870 |
|  |  |  |  |  | TRINITY_DN385145_c1_g1 | L-3-cyanoalanine synthase/ cysteine synthase | 2.2478 | -0.1961 |
|  |  |  |  |  | TRINITY_DN385145_c1_g3 | L-3-cyanoalanine synthase/ cysteine synthase | 2.5624 | -0.1762 |
|  |  |  |  |  | TRINITY_DN385145_c1_g2 | L-3-cyanoalanine synthase/ cysteine synthase | 2.6311 | 0.0577 |
| beta-Alanine metabolism | Uracil | Nucleotide and its derivates | 0.4907 | 0.2527 | TRINITY_DN339215_c0_g1 | glutamate decarboxylase | 2.2886 | 0.3310 |
|  |  |  |  |  | TRINITY_DN399803_c3_g7 | glutamate decarboxylase | 2.3775 | 0.3353 |
|  |  |  |  |  | TRINITY_DN353982_c0_g1 | glutamate decarboxylase | 2.4279 | 0.6460 |
| Histidine metabolism | 1-Methylhistidine | Amino acid derivatives | 3.2955 | 1.1997 | TRINITY_DN338271_c6_g1 | histidine decarboxylase | -3.2732 | -2.0874 |
| Pentose and glucuronate interconversions | Ribitol | Carbohydrates | 3.5365 | 1.7555 | TRINITY_DN382339_c1_g6 | UTP--glucose-1-phosphate uridylyltransferase | -2.6751 | -1.2040 |
| Purine metabolism | 2'-Deoxyguanosine | Nucleotide and its derivates | 0.4044 | 0.3217 | TRINITY_DN291157_c0_g1 | nucleoside-diphosphate kinase | -2.4589 | -1.0413 |
|  |  |  |  |  | TRINITY_DN317143_c11_g1 | adenosine kinase | -2.6611 | -1.0848 |
| Aminoacyl-tRNA biosynthesis | L-Asparagine | Amino acids | 2.1200 | 1.0940 | TRINITY_DN355582_c2_g3 | glutaminyl-tRNA synthetase | -4.6101 | -0.7478 |
|  | L-Alanine | Amino acids | 2.5374 | 1.4626 | TRINITY_DN298425_c0_g2 | seryl-tRNA synthetase | -2.9233 | -1.4806 |
|  |  |  |  |  | TRINITY_DN298319_c0_g1 | valyl-tRNA synthetase | -3.8694 | -0.4066 |
| Cyanoamino acid metabolism | L-Asparagine | Amino acids | 2.1200 | 1.0940 | TRINITY_DN385145_c0_g2 | L-3-cyanoalanine synthase/ cysteine synthase | 3.6006 | 0.72108 |
|  |  |  |  |  | TRINITY_DN367731_c3_g1 | L-3-cyanoalanine synthase/ cysteine synthase | 2.6683 | -0.1042 |
|  |  |  |  |  | TRINITY_DN352327_c0_g1 | L-3-cyanoalanine synthase/ cysteine synthase | 2.183 | -0.48275 |
|  |  |  |  |  | TRINITY_DN316068_c0_g1 | L-3-cyanoalanine synthase/ cysteine synthase | 2.5065 | -0.087038 |
|  |  |  |  |  | TRINITY_DN385145_c1_g1 | L-3-cyanoalanine synthase/ cysteine synthase | 2.2478 | -0.19614 |
|  |  |  |  |  | TRINITY_DN385145_c1_g3 | L-3-cyanoalanine synthase/ cysteine synthase | 2.5624 | -0.17621 |
|  |  |  |  |  | TRINITY_DN385145_c1_g2 | L-3-cyanoalanine synthase/ cysteine synthase | 2.6311 | 0.057714 |
| Pyrimidine metabolism | Uracil | Nucleotide and its derivates | 0.4907 | 0.2527 | TRINITY_DN291157_c0_g1 | nucleoside-diphosphate kinase | -2.4589 | -1.0413 |
|  |  |  |  |  | TRINITY_DN327045_c0_g1 | dihydroorotate dehydrogenase | #NAME? | -6.2681 |
| Glycerophospholipid metabolism | sn-Glycero-3-phosphocholine | Cholines | 2.0868 | 0.7719 | TRINITY_DN334074_c3_g1 | phosphatidylserine synthase 2 | -3.4202 | -1.2589 |


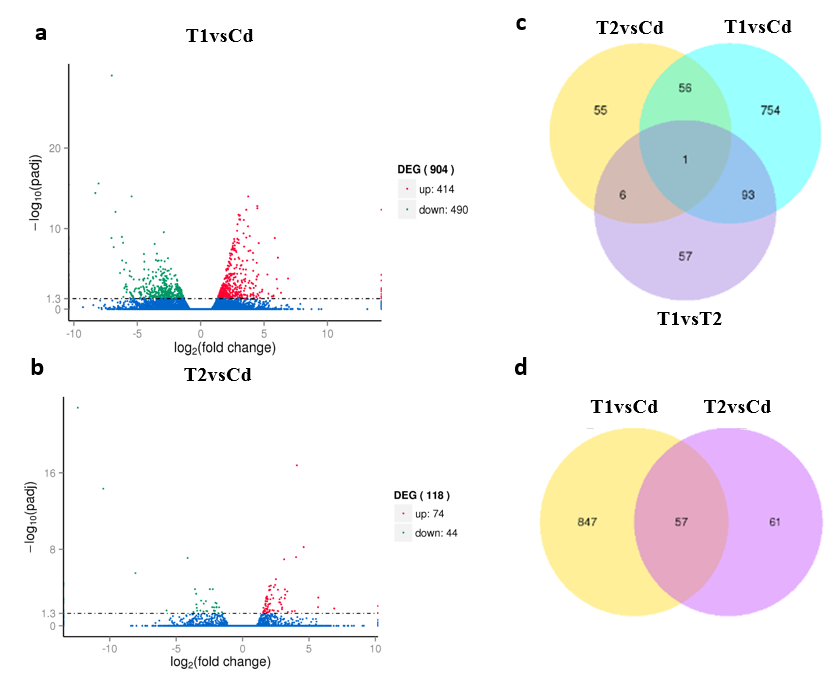


**Fig. S1.** Volcano Plots and Venn diagrams of significantly differentially expressed transcripts in the tall fescue roots with or without NO treatment under cadmium stress. (a) Volcano plot in T1vsCd. (b) volcano plot in T2vsCd. (c) Venn diagram analysis in different treatment. (d) Venn diagram analysis between T1vsCd and T2vsCd. Numbers indicate the number of transcripts with significant changes in expression under different conditions. Overlaps indicate the number of common transcripts differentially expressed, and numbers outside overlaps indicate the number of cultivar or subgroup specific transcripts differentially expressed. There were three regime, comprising Cd, T1, and T2. They respectively presented the tall fescue seedlings were cultivated in 1/2 Hoagland solution with 50 mg/L Cd^2+^ (CdCl_2_•2.5H_2_O) (Cd treatment), 1/2 Hoagland solution with 50 mg/L Cd^2+^ and 200 μM SNP (T1 treatment) and 1/2 Hoagland solution with 50 mg/L Cd^2+^, 200 μM L-NAME and 100 μM c-PTIO (T2 treatment). Each value is the mean of three replicates.


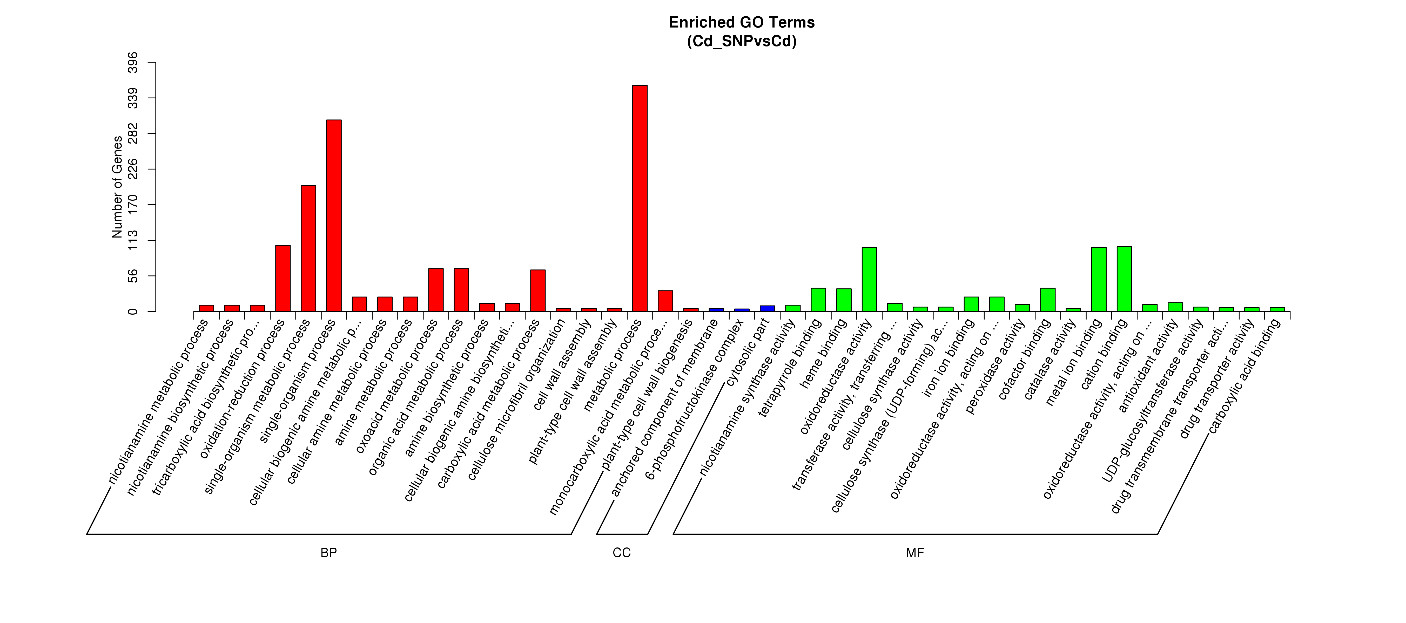
**Fig. S2** Histogram of the gene ontology classification analysis of the DEGs in response to the T1 treatment in tall fescue roots. The tall fescue seedlings were cultivated in 1/2 Hoagland solution with 50 mg/L Cd^2+^ (CdCl_2_•2.5H_2_O) (Cd treatment) and 1/2 Hoagland solution with 50 mg/L Cd^2+^ and 200 μM SNP (T1 treatment), respectively. Each value is the mean of three replicates.


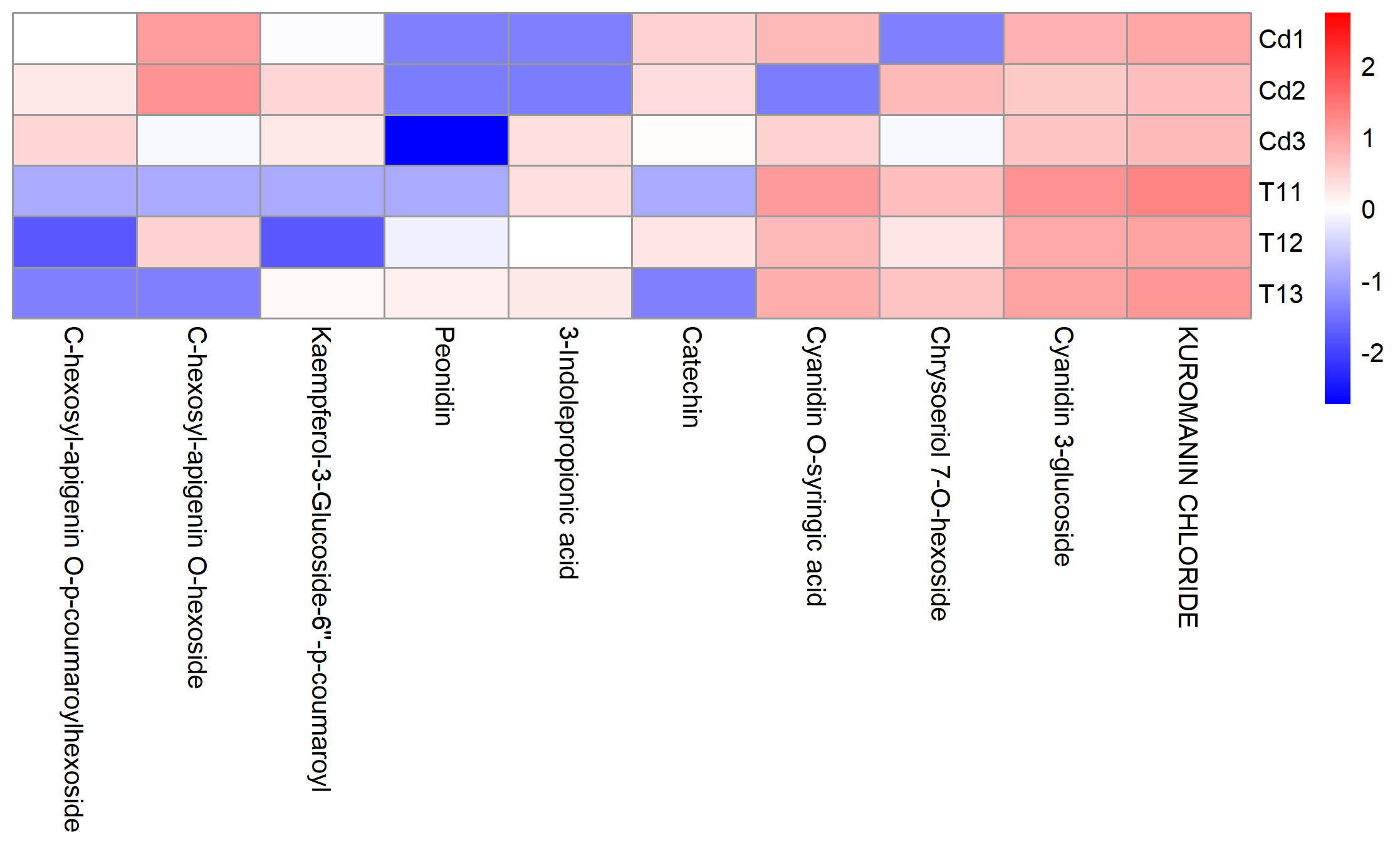


**Fig. S3.** The top 10 metabolites according to the VIP values in tall fescue under T1 treatment. The tall fescue seedlings were cultivated in 1/2 Hoagland solution with 50 mg/L Cd^2+^ (CdCl_2_•2.5H_2_O) (Cd treatment) and 1/2 Hoagland solution with 50 mg/L Cd^2+^ and 200 μM SNP (T1 treatment), respectively. Each value is the mean of three replicates.


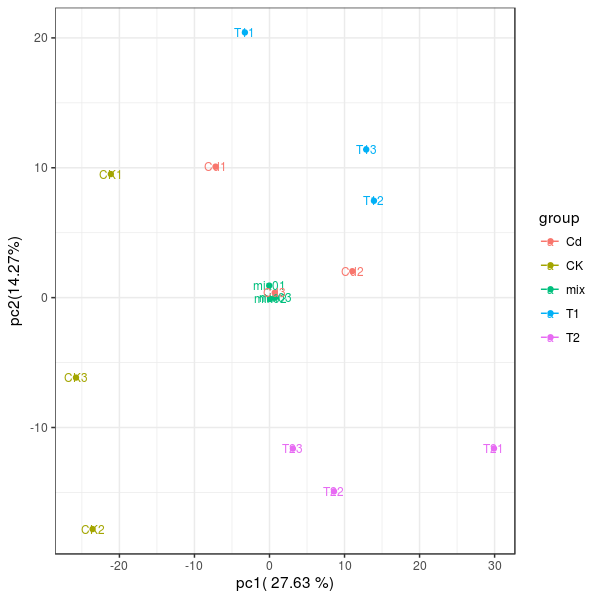


**Fig. S4** Principal component analysis (PCA) of the metabolite profiles in tall fescue roots. The analysis was performed on all the metabolites detected in tall fescue roots under different conditions. There were four treatment regimes in this study, including the control (CK), Cd treatment, T1 treatment, and T2 treatment, and each regime had three replicates. They respectively presented the tall fescue seedlings were cultivated in 1/2 Hoagland solution (control, CK), 1/2 Hoagland solution with 50 mg/L Cd^2+^ (CdCl_2_•2.5H_2_O) (Cd treatment), 1/2 Hoagland solution with 50 mg/L Cd^2+^ and 200 μM SNP (T1 treatment), and 1/2 Hoagland solution with 50 mg/L Cd^2+^, 200 μM L-NAME and 100 μM c-PTIO (T2 treatment).

**
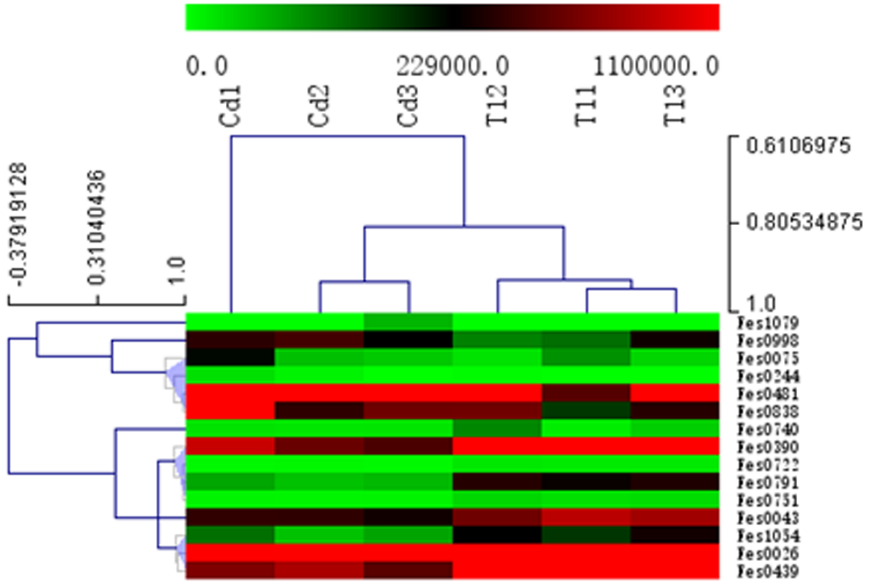
**

**Fig. S5** The hierarchical cluster analysis (HCA) of the differentially expressed metabolites selected from the integrated analysis between the T1 treatment and Cd treatment in tall fescue roots. The tall fescue seedlings were cultivated in 1/2 Hoagland solution with 50 mg/L Cd^2+^ (CdCl_2_•2.5H_2_O) (Cd treatment) and 1/2 Hoagland solution with 50 mg/L Cd^2+^ and 200 μM SNP (T1 treatment), respectively. Each value is the mean of three replicates.


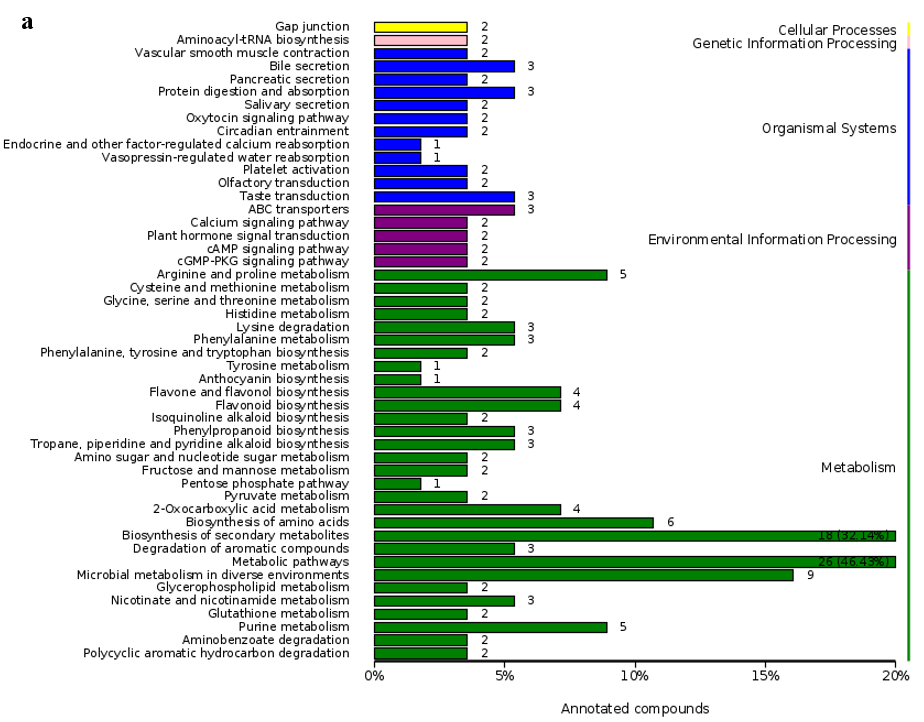

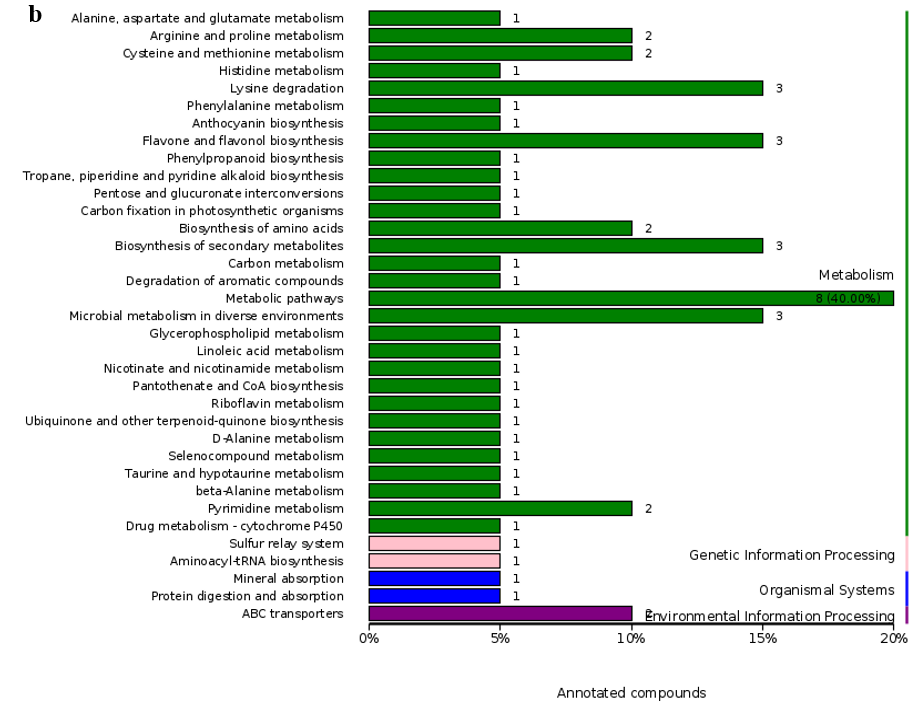


**Fig. S6** The distribution of metabolites in different KEGG pathways. (A) the metabolites in Cd vs Con. (B) the metabolites in T1 vs Cd. There were three regime, comprising Cd, T1 and T2. They respectively presented the tall fescue seedlings were cultivated in 1/2 Hoagland solution with 50 mg/L Cd^2+^ (CdCl_2_•2.5H_2_O) (Cd treatment), 1/2 Hoagland solution with 50 mg/L Cd^2+^ and 200 μM SNP (T1 treatment) and 1/2 Hoagland solution with 50 mg/L Cd^2+^ and 200 μM NG-nitro-L-Arg-methyl ester (L-NAME) and 100 μM 2-(4-carboxyphenyl)-4,4,5,5-tetramethylimidazoline-1-oxyl-3-oxide (cPTIO) (T2 treatment).


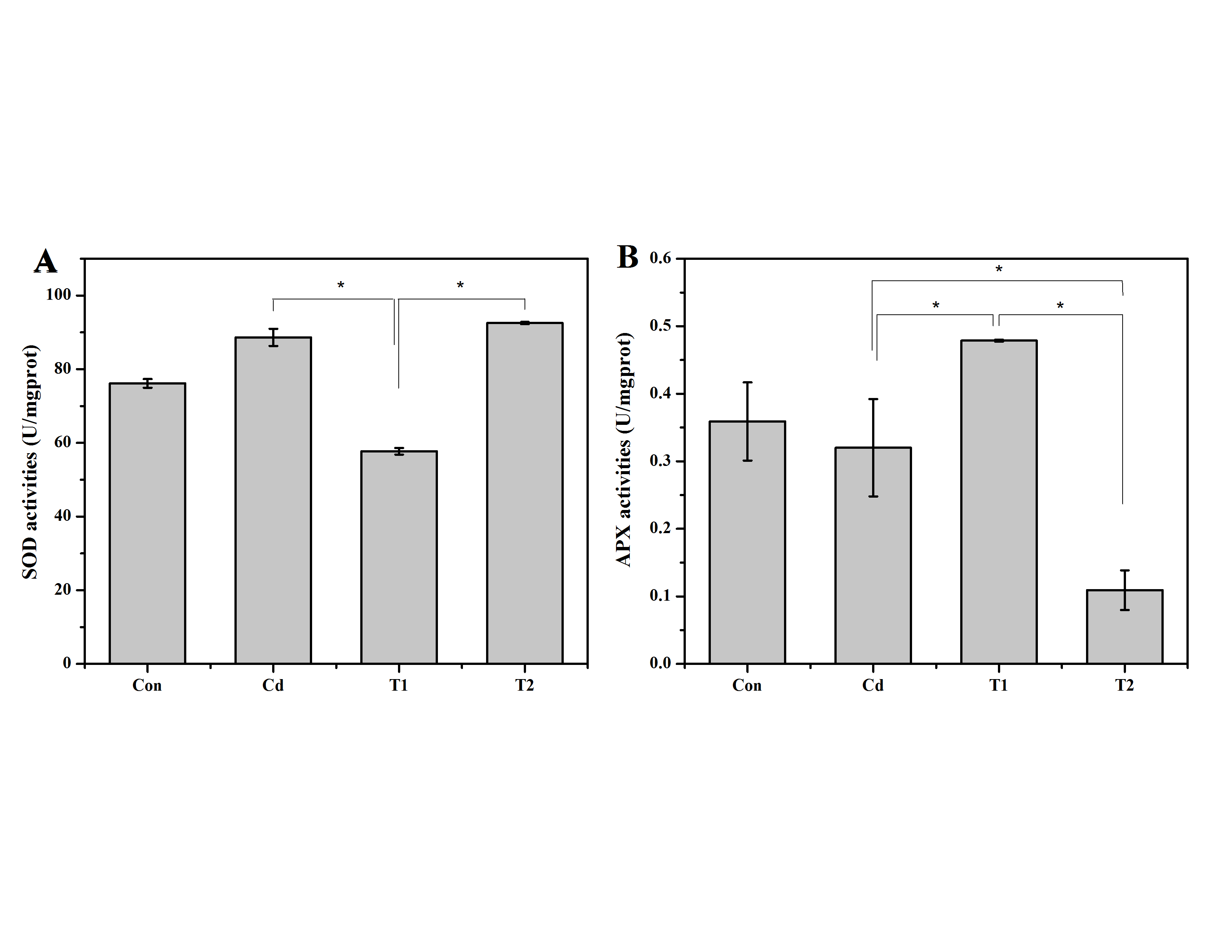


**Fig. S7** The activities of SOD (superoxide dismutase) and APX (Ascorbate peroxidase) in tall fescue roots. Values were given as means ± SD (n = 4). Data about Cd, T1 and T2 treatment were analyzed using one-way Analysis of Variance, followed by LSD test. Asterisks (*) indicate the significant difference at P < 0.05.

**Fig. S8** Correlations of expression level analyzed by RNA-Seq platform

(y axis) with data resulted from qRT-PCR (x axis).
